# High-Resolution Assessment of Terrestrial Carbon Storage and Restoration Benefits in China, 1990–2023

**DOI:** 10.1021/acs.est.6c00827

**Published:** 2026-08-31

**Authors:** Zhen Wu, Xianjin Huang, Michael E. Meadows

**Affiliations:** † School of Geography and Ocean Science, 12581 Nanjing University, Nanjing, Jiangsu 210023, China; ‡ The Key Laboratory of Carbon Neutrality and Territory Optimization, Ministry of Natural Resources, Nanjing, Jiangsu 210023, China; § Department of Geography and Resource Management, The Chinese University of Hong Kong, Shatin, 999077 Hong Kong SAR, China; ∥ Department of Environmental & Geographical Science, 37716 University of Cape Town, Rondebosch, Cape Town 7701, South Africa

**Keywords:** terrestrial carbon storage, remote sensing, machine learning, soil organic carbon, ecological restoration

## Abstract

Terrestrial ecosystems serve as critical carbon sinks, playing an important role in the global carbon cycle and climate regulation. However, previous estimates of carbon storage in China’s terrestrial ecosystems remain uncertain due to variability in data sources and methodological approaches. This study integrates multisource remote sensing data with extensive field-sampling data sets of carbon density, leveraging machine learning models to provide a comprehensive estimation of multiyear carbon storage and annual dynamics across multiple carbon pools. We estimated project-region carbon-stock changes using a scenario-based accounting framework. The total carbon storage, encompassing vegetation biomass and soil organic carbon, is estimated at 113.48 Pg C, with 20.4% in vegetation biomass and 79.6% in soil. Across land-use types, forests store the largest share, with 47.99 Pg C, followed by grasslands, croplands, barren lands, shrublands, and wetlands, which stored 32.32, 18.00, 14.65, 0.49, and 0.02 Pg C, respectively. National carbon stocks fluctuated over the study period but increased by 6.62 Pg C from 2014 to 2023. Scenario-based accounting indicates an additional baseline-adjusted project-region carbon-stock gain of 0.495 Pg C within major restoration regions during 2014–2023, equivalent to 0.050 Pg C yr^–1^. These results provide a high-resolution reconstruction of China’s terrestrial carbon stocks and clarify recent carbon-stock changes across ecosystems, regions, and major restoration areas.

## Graphical abstract

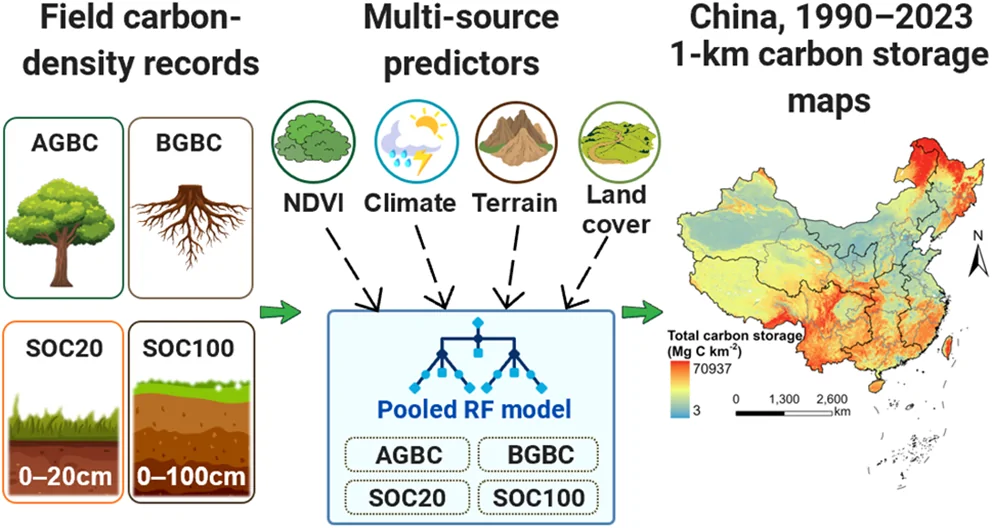

## 1 Introduction

Reducing carbon dioxide (CO_2_) emissions and enhancing terrestrial carbon sequestration are central challenges for mitigating regional and global climate change.^1^ Terrestrial ecosystems, including vegetation and soils, constitute major carbon reservoirs and play a critical role in regulating the global carbon cycle.^2–5^ During the 1980s and 1990s, terrestrial ecosystems offset approximately 10–60% of fossil fuel emissions,^6,7^ and more recent estimates suggest that global land ecosystems continue to absorb a substantial fraction of anthropogenic CO_2_ emissions.^8^ Meeting the Paris Agreement temperature targets therefore requires robust quantification of regional carbon stocks and their temporal dynamics.^9,10^ Because terrestrial carbon stocks are shaped by climate, land cover, ecosystem composition, soil properties, and human management, accurate assessments of their magnitude and spatial distribution are essential for carbon-cycle modeling, land-use planning, and climate-mitigation policy.^11–13^

China is the world’s largest annual emitter of CO_2_, and its terrestrial ecosystems play an important role in the regional and global carbon budget.^14,15^ Previous studies have suggested that carbon accumulation in China’s terrestrial biosphere offsets approximately 28–37% of fossil-fuel CO_2_ emissions, a proportion comparable to that reported for the United States (20–40%)^16^ and higher than that for Europe (12%).^17^ However, national-scale estimates of terrestrial carbon stocks in China remain highly uncertain. Vegetation carbon stock estimates range from 6.1 to 57.9 Pg C, whereas soil organic carbon stock estimates range from 37.27 to 185.7 Pg C, indicating substantial discrepancies among existing national terrestrial carbon-stock estimates for China (Table S8). This large variation mainly reflects differences in data sources, sampling periods, estimation methods, spatial resolution, ecosystem coverage, and carbon-pool definitions. Previous studies have used empirical or process-based models, literature syntheses, national inventory data, intensive field surveys, and carbon-transfer methods, each relying on different assumptions about carbon density, turnover, vegetation classification, soil depth, and spatial aggregation.^2,4,18–23^ In addition, differences in plant carbon content, soil depth, land-cover area, and inclusion or exclusion of belowground biomass can substantially affect national totals.^2,4^ These inconsistencies make direct comparison among existing estimates difficult and highlight the need for a high-resolution, ecosystem-wide, and methodologically consistent reconstruction of vegetation and soil carbon stocks across China.

Field observations remain the foundation for estimating vegetation biomass and soil organic carbon stocks, and many previous assessments have relied primarily on field sampling data^2,4,24^ (e.g., forest inventory records,^25–28^ or soil survey data^4,18,29,30^ ). Nevertheless, field-based estimates are often constrained by uneven sampling density, differences in measurement protocols, limited coverage of under-sampled ecosystems, and insufficient long-term monitoring.^31–33^ In addition, many studies have focused on individual ecosystem types, such as forests,^25,26,34^ grasslands,^35,36^ or croplands,^37,38^ rather than providing a consistent estimate across China’s major terrestrial ecosystems. Moreover, evidence from field sampling and model simulations indicates that carbon storage in China’s terrestrial ecosystems changes over time,^4^ while most estimates have primarily addressed specific, short time periods,^2,4,23,33^ limiting insights into the longer-term dynamics of carbon storage. Carbon stocks are highly heterogeneous across environmental gradients and land-cover mosaics, and coarse-resolution or ecosystem-specific estimates may obscure local hotspots of carbon storage, areas of rapid change, and spatial mismatches between restoration activities and carbon-stock responses. Therefore, high-resolution mapping is needed not only to improve national-scale estimates, but also to characterize regional heterogeneity, support ecosystem-specific carbon accounting, and evaluate carbon-stock changes within major land-management and ecological-restoration regions.

Remote sensing, gridded environmental data sets, field observations, and machine-learning approaches provide an opportunity to generate spatially continuous and temporally resolved estimates of terrestrial carbon stocks  .^39–41^ However, key gaps remain: previous estimates are often limited to specific time periods, single ecosystems, coarse spatial resolutions, or incomplete carbon-pool accounting; and few studies have reconstructed annual, high-resolution carbon-stock dynamics across vegetation and soil pools for all major terrestrial ecosystems in China over multiple decades. Addressing these gaps is essential for understanding how China’s terrestrial carbon stocks have changed over time and for providing a more robust empirical basis for carbon-cycle assessment and land-based mitigation planning.

In this study, we integrated 6,793 field-sampled carbon-density records from published sources with remote-sensing and environmental predictors to reconstruct annual carbon-stock dynamics across China’s terrestrial ecosystems from 1990 to 2023 at a spatial resolution of 1-km. The assessment covered croplands, forests, grasslands, shrublands, wetlands, and barren lands, and included aboveground biomass carbon, belowground biomass carbon, and soil organic carbon at depths of 0–20 cm and 0–100 cm. Specifically, our objectives were to (1) generate a consistent high-resolution reconstruction of vegetation and soil carbon stocks across China’s terrestrial ecosystems; (2) quantify the spatial distribution and temporal dynamics of carbon stocks from 1990 to 2023; (3) compare our estimates with previous national-scale assessments and clarify the role of differences in accounting boundaries; and (4) evaluate carbon-stock changes within major ecological-restoration regions using a scenario-based accounting framework. The resulting data set provides an updated and spatially explicit assessment of China’s terrestrial carbon stocks and offers a quantitative basis for carbon-cycle modeling, ecosystem management, and climate-mitigation planning.

## 2 Materials and Methods

### 2.1 Data Sources and Predictor Variables

Predictor variables were selected to represent major environmental, spatial, temporal, and ecosystem controls on terrestrial carbon density. Annual Normalized Difference Vegetation Index (NDVI) was used as a proxy for vegetation greenness and productivity.^27^ Mean annual temperature (MAT) and mean annual precipitation (MAP) represented broad climatic constraints on plant growth and soil carbon processes.^4^ Elevation and slope were included to represent topographic controls on vegetation distribution, microclimate, and soil development.^42^ Latitude and year were included as continuous spatial and temporal covariates, respectively. Land-cover type and carbon-pool type were included as nominal categorical predictors to distinguish ecosystem types and carbon pools.

This study calculated the annual NDVI for China from 1990 to 2023 using Landsat 5/7/8/9 data via the Google Earth Engine (GEE) platform, with a spatial resolution of 30 m. Elevation and slope data for the 2000s were calculated from the GEE platform using the Shuttle Radar Topography Mission (SRTM) digital elevation data, also with a 30-m spatial resolution. Because topography is effectively static over the study period, a single topographic layer was used for all years. Annual land-cover data for 1990–2023 were obtained from the China Land Cover Data set (CLCD) at 30-m spatial resolution, which can be downloaded from the open data platform at 10.5281/zenodo.15853565.^43,44^ Annual temperature and precipitation data for 1990–2023 were sourced from the National Earth System Science Data Center (https://www.geodata.cn), with a spatial resolution of approximately 1,000 m.^45^ Boundaries of the major ecological restoration projects were obtained from the National Ecosystem Science Data Center, National Science & Technology Infrastructure of China (http://www.nesdc.org.cn) (Figure S7; Table S9). All predictor layers were harmonized to the 1-km prediction grid before model fitting and gridded prediction. For carbon-stock accounting, we retained cropland, forest, shrubland, grassland, wetland, and barren land as main terrestrial ecosystem classes.

Field-sampled carbon-density records for China’s terrestrial ecosystems during 2000–2014 were obtained from the Chinese National Ecosystem Science Data Center (http://www.nesdc.org.cn).^4,46^ We retained records with complete information on geographic coordinates, sampling year, carbon-pool type, land-cover type, and measured carbon density. This screening yielded 6,793 field-measured carbon-density records. The records covered four carbon pools: aboveground biomass carbon density (AGBC), belowground biomass carbon density (BGBC), soil organic carbon density at 0–20 cm depth (SOC20), and soil organic carbon density at 0–100 cm depth (SOC100). The data set covered cropland, forest, grassland, shrubland, wetland, and barren land.

### 2.2 Carbon Density Modeling and Gridded Prediction

We developed a pooled Random Forest model to predict carbon density from the selected predictor variables. Continuous predictors included annual NDVI, MAT, MAP, elevation, slope, latitude, and year. Land-cover type and carbon-pool type were treated as nominal categorical predictors. The calibration records from 2000 to 2014 were used for model development. The internal model-development procedure used a 70/30 split for training and internal testing, repeated 100 times. These internal metrics were used for model selection rather than as the primary evidence of map accuracy. The Random Forest model achieved an internal *R* ^2^ of 0.763, with MAE and RMSE values of 1.2 and 2.4 kg C m^–2^, respectively. The final model used for gridded prediction was then fitted using the full calibration data set.

Annual gridded predictions were generated from 1990 to 2023. For each year, the fitted pooled Random Forest model was applied in four carbon-pool-conditioned prediction runs. In each run, the carbon-pool category was fixed to AGBC, BGBC, SOC20, or SOC100, while the corresponding annual environmental, land-cover, spatial, and temporal predictors were supplied. This procedure produced annual 1-km carbon-density rasters for AGBC, BGBC, SOC20, and SOC100 across cropland, forest, grassland, shrubland, wetland, and barren land (Figure 1). This carbon density data set is available for public download and can be accessed from 10.5281/zenodo.21372793.^47^

**Figure 1:**
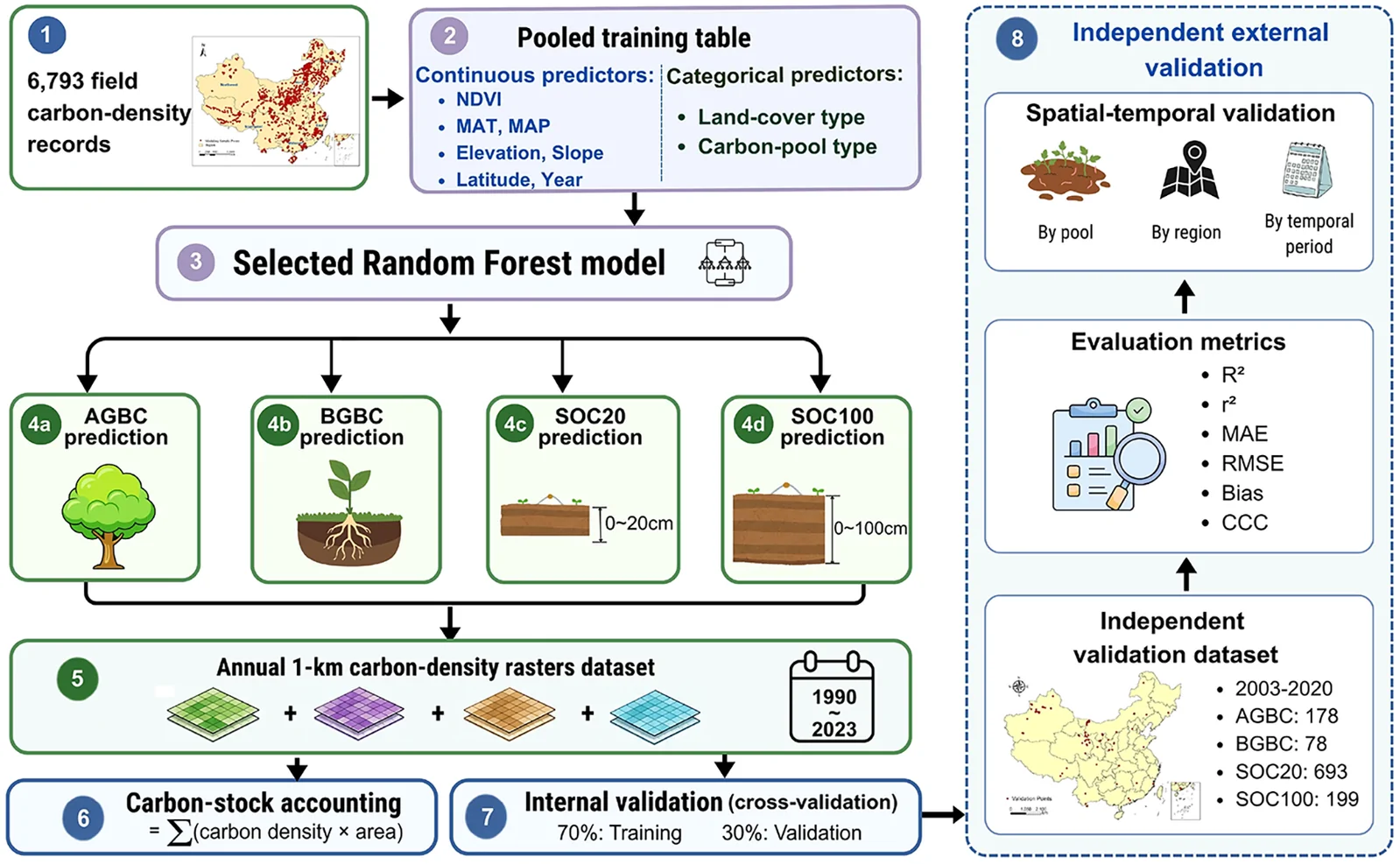
Workflow for pooled Random Forest model training, independent validation, carbon-pool-conditioned prediction, and carbon-stock accounting.

### 2.3 Independent Validation and Diagnostic Analyses

We independently evaluated the gridded carbon-density predictions using 1,148 field observations compiled from peer-reviewed literature. These records covered 2003–2020 and were not used for model training, internal testing, or model selection. The validation data set included 178 AGBC records, 78 BGBC records, 693 SOC20 records, and 199 SOC100 records. For each validation record, the predicted carbon density was extracted from the annual 1-km raster corresponding to the same year, geographic location, and carbon-pool category. AGBC, BGBC, SOC20, and SOC100 observations were therefore evaluated against their corresponding pool-specific prediction rasters.

Model performance was evaluated using the coefficient of determination (*R* ^2^), squared Pearson correlation (*r* ^2^), mean absolute error (MAE), root-mean-square error (RMSE), mean bias error (Bias), and Lin’s concordance correlation coefficient (CCC). The formulas for these metrics are provided in the Supporting Information. To reduce the influence of repeated observations from the same spatial locations, we conducted a sensitivity analysis after aggregating records by site ID, year, and carbon-pool category (Table S1).

We estimated uncertainty intervals for the overall validation metrics using site-ID-cluster bootstrap resampling with 1,000 iterations. In each iteration, site IDs were resampled with replacement, and all validation records associated with selected site IDs were retained as clusters. The 2.5th and 97.5th percentiles of the bootstrap distributions were used to define 95% bootstrap intervals (Table S2). Practical bootstrap uncertainty intervals for national and regional carbon-stock estimates are reported separately in Table S5.

Validation statistics were also calculated by carbon pool, region, and temporal period (Tables S1, S3, and S4). These stratified statistics were interpreted as diagnostic summaries of model performance and sample support rather than as design-unbiased estimates of map accuracy for each stratum, particularly where the number of unique site IDs was limited. To assess temporal transferability, we separately evaluated validation records from the calibration-overlap period (2003–2014) and the postcalibration period (2015–2020) (Table S4).

### 2.4 Carbon Storage Estimates

The total carbon storage of terrestrial ecosystems is calculated as the sum of vegetation biomass carbon storage and soil organic carbon storage. Vegetation biomass carbon storage includes both aboveground and belowground biomass carbon, while soil organic carbon storage refers to carbon stock within the 0–100 cm soil depth.^4^ SOC20 was analyzed separately to characterize topsoil carbon patterns but was not added to SOC100 in the total carbon-stock calculation because SOC20 is already included within the 0–100 cm SOC pool.^4^ Soil carbon below 1-m was outside the accounting boundary of this study, consistent with the available field observations and reconstructed soil carbon pools.^2^

For cropland, vegetation biomass carbon was excluded from the national total because cropland biomass is highly seasonal and is regularly harvested under intensive management. Cropland carbon stock was therefore represented by soil organic carbon only.^2,4^ Litter carbon was also excluded because it was not consistently represented in the field observations or reconstructed carbon pools. These accounting choices improve internal consistency but limit direct comparability with studies that include standing cropland biomass, litter, dead wood, or soil carbon below 1-m. Previous research suggests that cropland vegetation carbon stock and litter carbon storage are approximately 2.0 Pg C^18^ and 0.52 Pg C,^48^ respectively.

Carbon density was converted to carbon stock by multiplying pool-specific carbon density by the corresponding ecosystem area and converting units to Pg C. The analysis covered 34 provincial administrative regions of China, including Taiwan, Hong Kong, and Macao. The detailed methodology for carbon storage calculation is outlined as follows:

1 $${Veg}_{\left(stock\right)}=\sum_{i=1}^{n}\left({Density}_{AGBC,i}+{Density}_{BGBC,i}\right)\times S_{i}$$

2 $${SOC}_{\left(stock\right)}={SOC100}_{\left(stock\right)}=\sum_{i=1}^{n}{Density}_{SOC100,i}\times S_{i}$$

3 $${\mathrm{SOC}}_{\left(\mathrm{topsoil}\_\mathrm{stock}\right)}={\mathrm{SOC}20}_{\left(\mathrm{stock}\right)}=\sum_{i=1}^{n}{\mathrm{Density}}_{\mathrm{SOC}20,i}\times S_{i}$$

4 $${\mathrm{Ecosystem}}_{\mathrm{stock}}={\mathrm{Veg}}_{\left(\mathrm{stock}\right)}+{\mathrm{SOC}}_{\left(\mathrm{stock}\right)}$$

Where, *n* represents the number of ecosystems, *S*_*i*_ is the area of ecosystem *i*, Density_AGBC,*i*_, Density_BGBC,*i*_, Density_SOC20,*i*_ and Density_SOC100,*i*_ denote aboveground biomass carbon density, belowground biomass carbon density, soil organic carbon density at 0–20 cm depth, and soil organic carbon density at 0–100 cm depth, respectively. Veg_(stock)_ and SOC_(stock)_ represent vegetation biomass carbon stock and soil organic carbon stock, respectively.

For spatial mapping, grid-cell carbon stock was calculated for each 1-km grid cell by multiplying land-cover-specific carbon density by the corresponding land-cover area within the grid cell and then summing across the six land-cover classes. Grid-cell carbon stocks are reported in Mg C km^–2^ for map display.

### 2.5 Baseline-Adjusted Accounting of Carbon-Stock Gains within Ecological Restoration Project Regions

We used a scenario-based accounting framework to estimate baseline-adjusted carbon-stock gains within ecological restoration project regions. This analysis covered seven national ecological restoration programs (Table S9) and used the 1-km annual carbon-stock rasters for 2014 and 2023. The results were summarized at provincial and national scales. First, we calculated gross carbon-stock changes within project-region footprints as^49^

5 $${\Delta C}_{\mathrm{project}}={\mathrm{CS}}_{\mathrm{project}\_2023}-{\mathrm{CS}}_{\mathrm{projec}t_{2014}}$$

where ΔC_project_ is the gross carbon-stock change within a project region from 2014 to 2023, CS_project_2023_ and CS_project_2014_ are the corresponding total carbon stocks in 2023 and 2014, respectively.

We then estimated a baseline-adjusted project-region carbon-stock gain by comparing carbon-stock changes within project regions with an accounting baseline derived from nonproject areas within the same province. These two accounting cases are referred to as the *project scenario* and the *baseline scenario*, respectively.^49^ The baseline-adjusted project-region carbon-stock gain was estimated as

6 $${\Delta C}_{\mathrm{BA}}={\Delta C}_{\mathrm{project}}-{\Delta C}_{\mathrm{baseline}}$$

where ΔC_BA_ represents the baseline-adjusted project-region carbon-stock gain, ΔC_project_ represents the carbon stock increase under the project scenario, and ΔC_baseline_ represents the increase under the baseline scenario. The value of ΔC_baseline_ was calculated as follows

7 $${\Delta C}_{\mathrm{baseline}}={\mathrm{CS}}_{\mathrm{base},2023}-{\mathrm{CS}}_{\mathrm{base},2014}$$

8 $${\mathrm{CS}}_{\mathrm{base},2023}=\sum_{i,k}\left({\mathrm{Veg}}_{\mathrm{base},2023,k}+{\mathrm{SOC}}_{\mathrm{base},2023,k}\right)\times A_{\mathrm{project},2023,i,k}$$

9 $${\mathrm{CS}}_{\mathrm{base},2014}=\sum_{i,k}{({\mathrm{Veg}}_{\mathrm{base},2014,k}+\mathrm{SOC}}_{\mathrm{base},2014,k})\times A_{\mathrm{pr}\mathrm{oject},2014,i,k}$$

CS_base,2023_ and CS_base,2014_ represent carbon stocks in 2023 and 2014, respectively, under the baseline scenario. We first identified areas with and without project implementation in each province, then calculated the average vegetation and soil carbon densities in nonproject areas. As a practical accounting baseline, we used the average vegetation and soil carbon densities of nonproject areas within the same province to approximate the baseline condition for project areas. Veg_base,2023,*k*_, Veg_base,2014,*k*_, SOC_base,2023,*k*_ and SOC_base,2014,*k*_ denote vegetation and soil carbon densities, respectively, in project areas under the baseline scenario for 2023 and 2014. *A*_project,2023,*i*,*k*_ and *A*_project,2014,*i*,*k*_ indicate the land cover area of project *i* in province *k* for 2023 and 2014.

Consistent with the national accounting boundary, cropland biomass was excluded from the GGP accounting in this study.^4^ To account for soil carbon retention (SCR) associated with the Grain for Green Program (GGP), SCR was used as a project-specific accounting indicator.^49^ SCR was calculated at a 1-km grid resolution and then aggregated to provincial and national scales. GGP-related soil carbon retention was calculated as follows

10 $$C_{\mathrm{GGP},\mathrm{SCR}}=\sum {\mathrm{RSCR}}_{k}\times A_{k}\times Y$$

RSCR_*k*_ represents the rate of soil carbon retention resulting from erosion control attributed to the GGP in province *k*, 0.029 kg C m^–2^ yr^–1^ for Shanxi, Shaanxi, Gansu, Inner Mongolia, and Qinghai, and 0.033 kg C m^–2^ yr^–1^ for all other provinces.^49^ *A*_*k*_ denotes the area of cropland converted to forest in province *k*, and *Y* is the project duration in years. *A*_*k*_ was converted to m^2^ before multiplication, and the resulting carbon mass was converted from kg C to Pg C.

In addition, to test the sensitivity of the project-region accounting results to the choice of reference year, we repeated the carbon-stock change calculation using a local three-year reference window centered on 2014. For each year, the seven project-region raster records were merged by pixel ID to form a union footprint, with overlapping project areas counted only once. Vegetation biomass carbon, soil organic carbon, and total carbon stock were summed separately within the union footprint for 2013, 2014, 2015, and 2023. Carbon-stock changes were then calculated as the difference between 2023 and each reference year from 2013 to 2015. The resulting 2013–2023, 2014–2023, and 2015–2023 changes were compared to evaluate whether the direction and magnitude of the estimates were sensitive to the choice of reference year (Table S6).

## 3 Results

### 3.1 Independent Validation

The independent validation data set included 1,148 field records that were not used for model training, model comparison, or model selection. Across all validation records, the gridded carbon-density predictions agreed well with field observations, with an *R* ^2^ of 0.698, an *r* ^2^ of 0.720, an MAE of 1.506 kg C m^–2^, an RMSE of 2.442 kg C m^–2^, and a mean bias of 0.120 kg C m^–2^ (Figure 2; Tables S1–S2). Site-ID-cluster bootstrap intervals were 0.637–0.741 for *R* ^2^, 1.212–1.881 kg C m^–2^ for MAE, and 1.870–3.088 kg C m^–2^ for RMSE, indicating that the overall validation statistics were relatively stable when spatial site IDs were resampled as clusters. Additional validation metrics are reported in Table S1.

**Figure 2:**
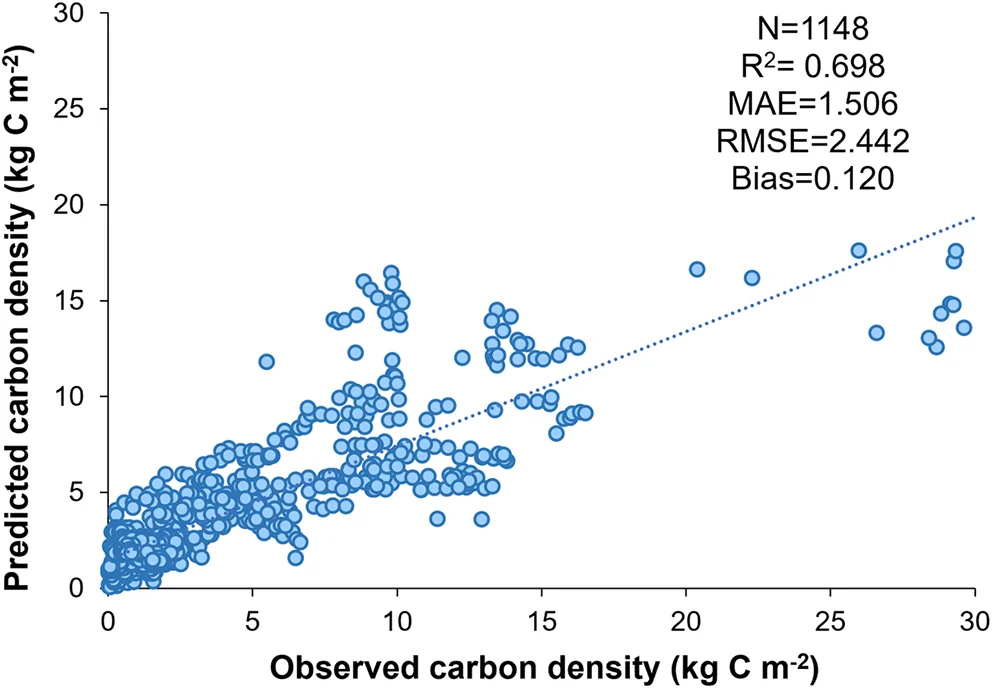
Independent validation of gridded carbon-density predictions. Points show observed and predicted carbon density for 1,148 independent field records.

Aggregating repeated validation records by site ID, year, and carbon-pool category produced similar predictive agreement, with an *R* ^2^ of 0.693, although the RMSE increased to 2.790 kg C m^–2^ (Table S1). This suggests that repeated observations did not drive the overall model agreement, but they did affect the estimated magnitude of prediction errors.

Temporal validation indicated that the model retained predictive skill beyond the main calibration-supported period. For validation records from 2003 to 2014, the model achieved an *R* ^2^ of 0.705 and an RMSE of 2.592 kg C m^–2^. For the postcalibration subset from 2015 to 2020, performance remained comparable, with an *R* ^2^ of 0.668 and an RMSE of 2.068 kg C m^–2^ (Table S4). These results provide partial support for the post-2014 reconstruction. However, the 1990–1999 hindcast and the 2021–2023 estimates remain less directly constrained by field observations and should be interpreted as higher-uncertainty temporal reconstructions.

Validation performance differed among carbon pools. AGBC, SOC20, and SOC100 achieved *R* ^2^ values of 0.520, 0.595, and 0.563, respectively. BGBC showed weaker external agreement, with an *R* ^2^ of −0.364 and an RMSE of 1.155 kg C m^–2^ (Table S1). This weaker performance likely reflects the limited number of BGBC validation records and the greater uncertainty associated with belowground biomass allocation. BGBC-related results are therefore interpreted more cautiously than AGBC and SOC results. Region-stratified validation statistics are reported in the Supporting Information (Table S3).

### 3.2 Carbon Storage and Spatial Distribution in 2023

In 2023, China’s terrestrial ecosystems stored an estimated 113.48 Pg C in vegetation biomass and soil organic carbon across croplands, forests, grasslands, shrublands, wetlands, and barren lands. Vegetation biomass carbon and soil organic carbon stocks were estimated at 23.15 and 90.33 Pg C, respectively, in 2023 (Table S7). Soil organic carbon was therefore nearly 3.9 times larger than vegetation biomass carbon, highlighting the dominance of soils in China’s terrestrial carbon stock. Forests stored the largest total carbon stock among ecosystem types, followed by grasslands, croplands, barren lands, shrublands, and wetlands. The corresponding carbon stocks were 47.99, 32.32, 18.00, 14.65, 0.49, and 0.02 Pg C, respectively. The spatial distribution of 1-km grid-cell terrestrial carbon stock showed higher values in northeastern and southern China and lower values toward the northwest (Figure 3). This spatial pattern was consistent with broad differences in climate, vegetation cover, and ecosystem composition across China.^19^

**Figure 3:**
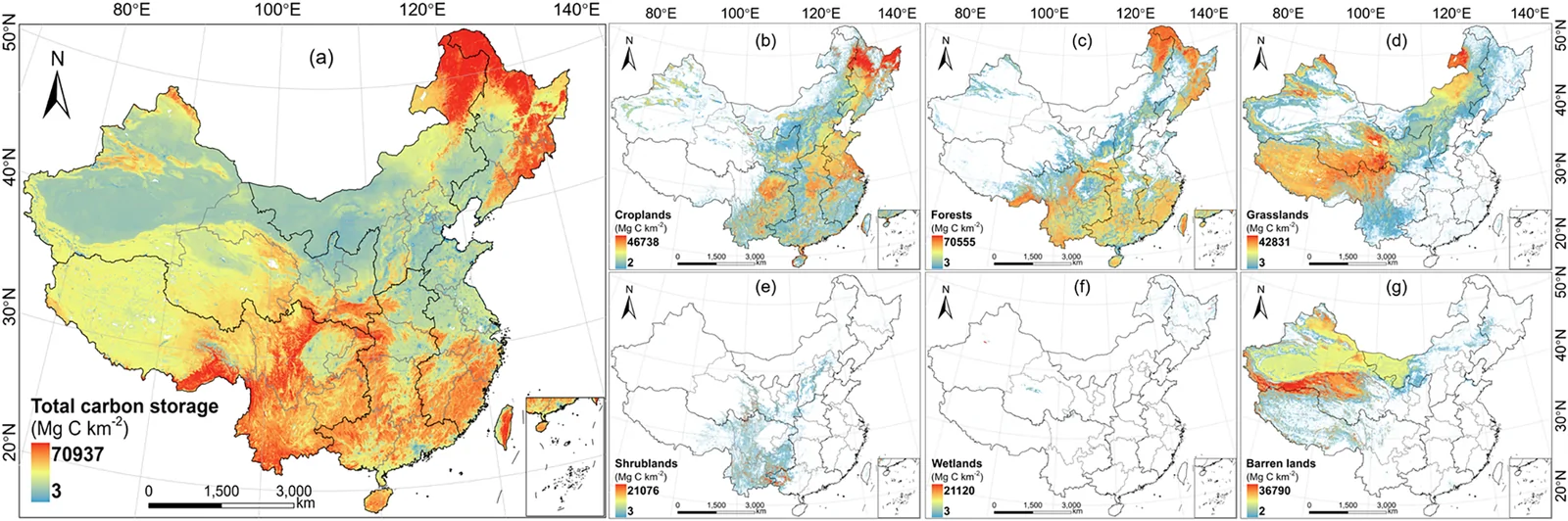
Spatial distribution of 1-km grid-cell terrestrial carbon stock in China in 2023. Panels show (a) all terrestrial ecosystems, (b) croplands, (c) forests, (d) grasslands, (e) shrublands, (f) wetlands, and (g) barren lands. Values are expressed as Mg C km^–2^. Map lines delineate the study area and do not necessarily represent accepted national boundaries.

Vegetation biomass carbon was concentrated mainly in forests, which stored 16.25 Pg C, followed by grasslands (4.54 Pg C), barren lands (2.20 Pg C), shrublands (0.15 Pg C), and wetlands (0.007 Pg C). Soil organic carbon exceeded vegetation biomass carbon in all major ecosystem types and showed strong regional variation. Soil organic carbon stocks in croplands, forests, grasslands, shrublands, wetlands, and barren lands were approximately 18.00, 31.74, 27.78, 0.34, 0.02, and 12.45 Pg C, respectively (Figure 4).

**Figure 4:**
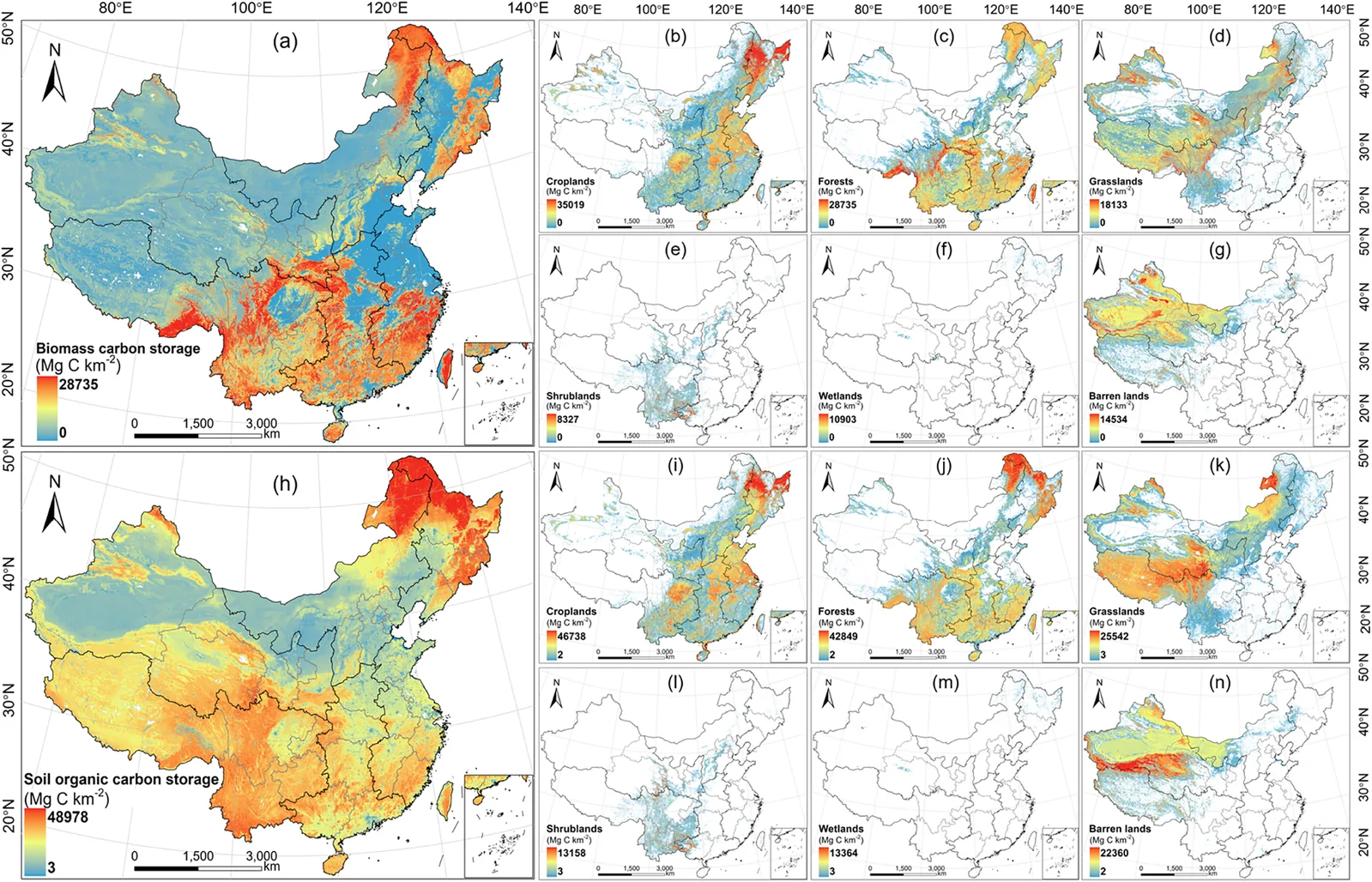
Spatial distribution of 1-km grid-cell vegetation biomass carbon stock and soil organic carbon stock across China’s terrestrial ecosystems in 2023. Panels (a–g) show vegetation biomass carbon stock and panels (h–n) show soil organic carbon stock for total terrestrial ecosystems, croplands, forests, grasslands, shrublands, wetlands, and barren lands, respectively. Values are expressed as Mg C km^–2^. Map lines delineate study areas and do not necessarily depict accepted national boundaries.

At the regional ecosystem scale, vegetation biomass carbon and soil organic carbon showed distinct component-specific patterns. Area-normalized forest vegetation biomass carbon stock was highest in Midsouth and Northeast China, reaching 3.73 and 2.91 kg C m^–2^, respectively, whereas area-normalized grassland vegetation biomass carbon stock was highest in Southwest and North China, at 0.70 and 0.67 kg C m^–2^, respectively. Area-normalized soil organic carbon stock was relatively high in Northeast, Midsouth, and Southwest China. Area-normalized forest soil organic carbon stock was highest in Northeast China, reaching 7.04 kg C m^–2^, whereas area-normalized grassland and shrubland soil organic carbon stocks were highest in Southwest China, at 5.33 and 0.10 kg C m^–2^, respectively.

At the regional scale, Northeast China had the highest area-normalized terrestrial carbon stock in 2023, at 15.37 kg C m^–2^, followed by Southwest China (14.73 kg C m^–2^), Midsouth China (13.94 kg C m^–2^), and East China (11.95 kg C m^–2^). These values exceeded the national average of 11.91 kg C m^–2^. Northwest and North China had lower area-normalized terrestrial carbon stocks, at 9.24 and 9.70 kg C m^–2^, respectively. At the provincial level, Yunnan, Heilongjiang, and Fujian had the highest area-normalized terrestrial carbon stocks, reaching 18.38, 17.85, and 17.26 kg C m^–2^, respectively, whereas Macao had the lowest value, at 1.26 kg C m^–2^ (Figure 5).

**Figure 5:**
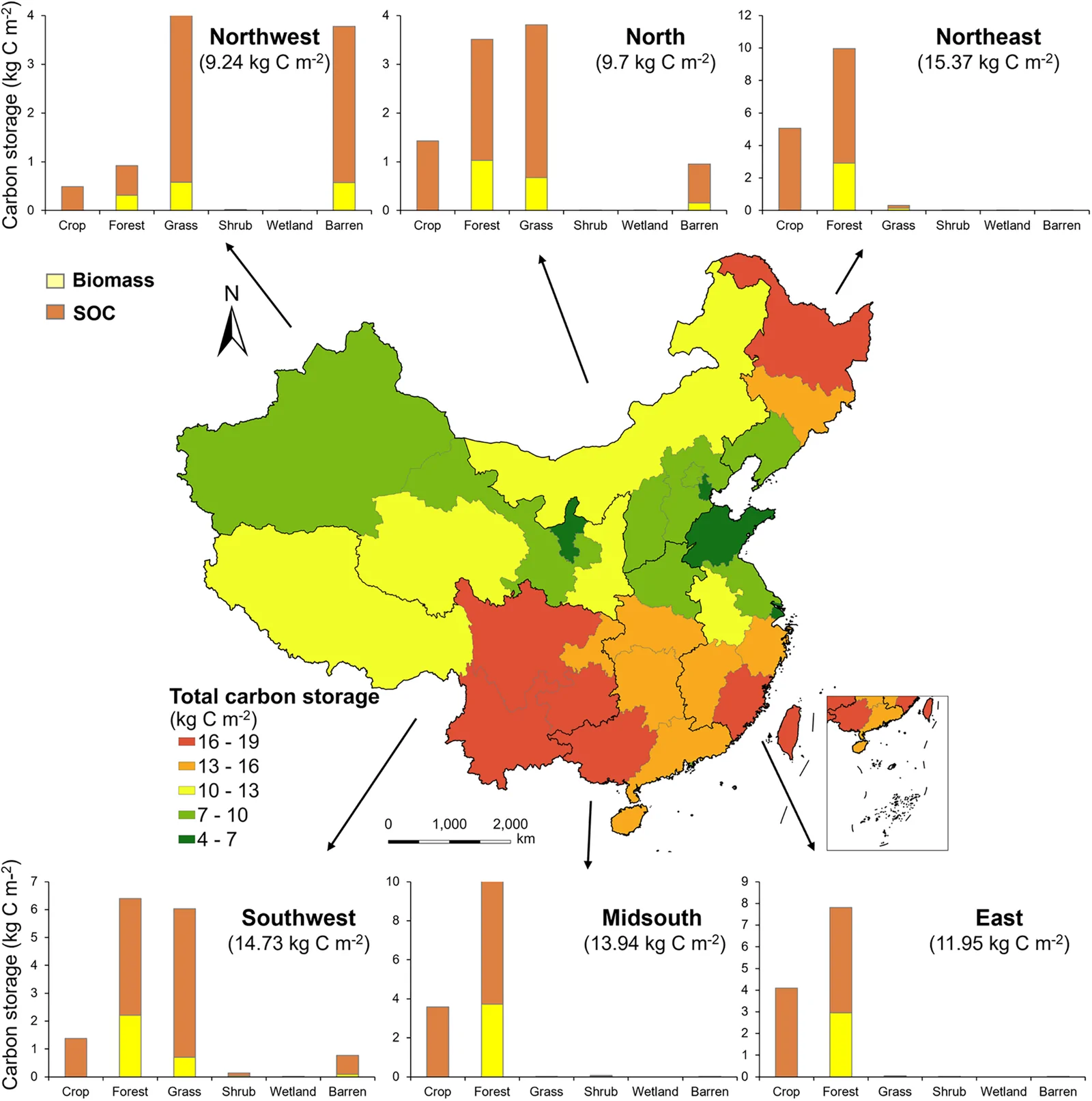
Regional and provincial area-normalized terrestrial carbon stocks in China in 2023. The map shows provincial area-normalized terrestrial carbon stocks, and inset bar charts summarize vegetation biomass carbon and soil organic carbon by ecosystem type within each region. Map lines delineate the study area and do not necessarily represent accepted national boundaries.

At the provincial level, area-normalized soil organic carbon stock exceeded area-normalized vegetation biomass carbon stock in all provincial-level administrative regions, indicating the widespread dominance of soil carbon in provincial area-normalized carbon stocks (Figure 6). Area-normalized vegetation biomass carbon stock was highest in Fujian and Yunnan, reaching 5.61 and 4.99 kg C m^–2^, respectively. Area-normalized soil organic carbon stock was highest in Heilongjiang, at 14.46 kg C m^–2^.

**Figure 6:**
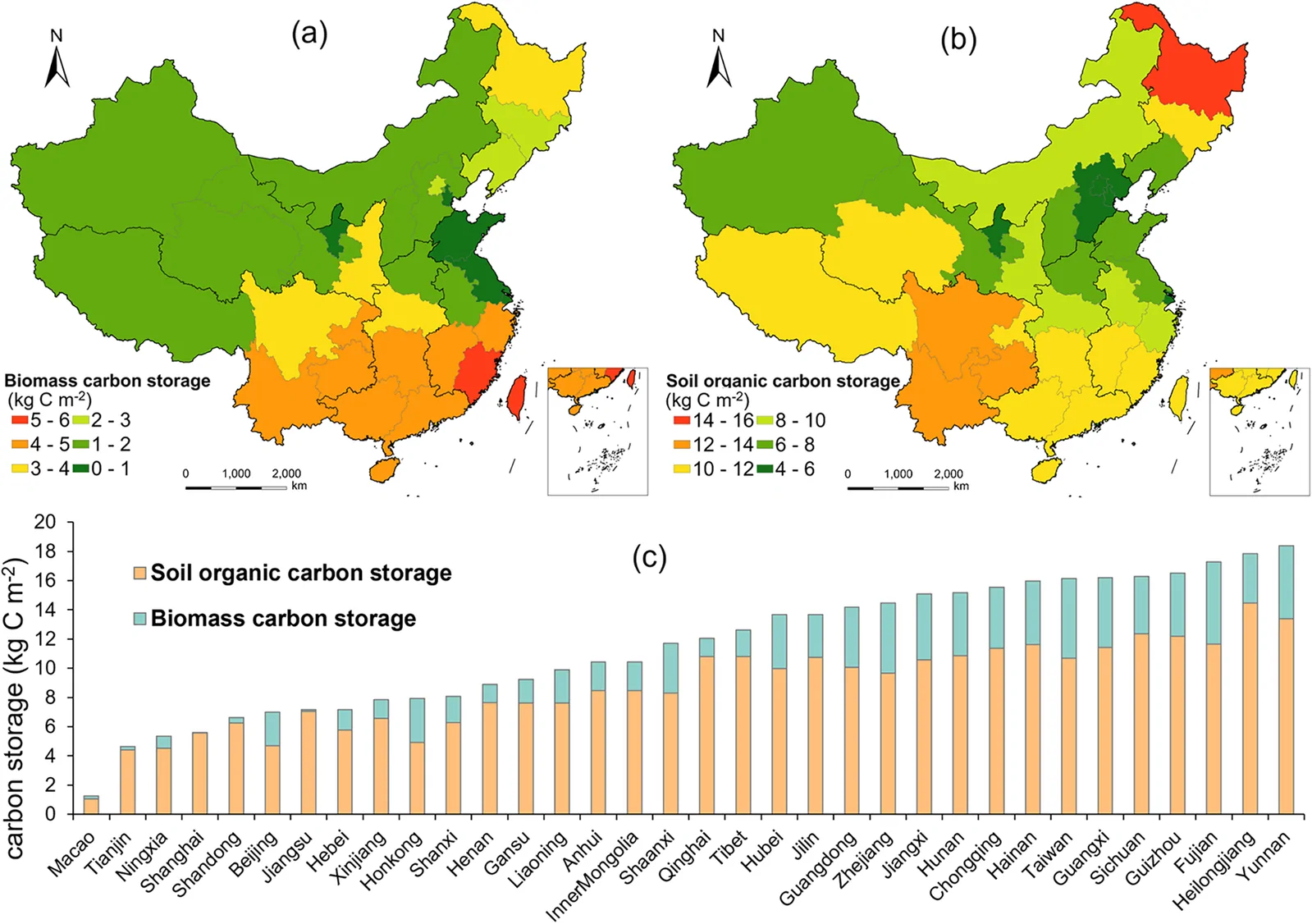
Area-normalized vegetation biomass carbon stock and soil organic carbon stock across provincial-level administrative regions in 2023. Panels show (a) area-normalized vegetation biomass carbon stock, (b) area-normalized soil organic carbon stock, and (c) provincial area-normalized carbon stock by component, shown as stacked bars. Map lines delineate the study area and do not necessarily represent accepted national boundaries.

### 3.3 Spatiotemporal Dynamics of Carbon Storage

Total carbon stock in China’s terrestrial ecosystems fluctuated from 1990 to 2023, with an early decline, a subsequent recovery, a short decline around 2012–2014, and a renewed increase after 2014 (Figures 7,S1–S2). National carbon stock declined by 8.50 Pg C from 1990 to 2002, increased by 6.58 Pg C from 2002 to 2012, and then decreased briefly from 2012 to 2014. From 2014 to 2023, national carbon stock increased by 6.62 Pg C, equivalent to approximately 6% of the 2023 national terrestrial carbon stock.

**Figure 7:**
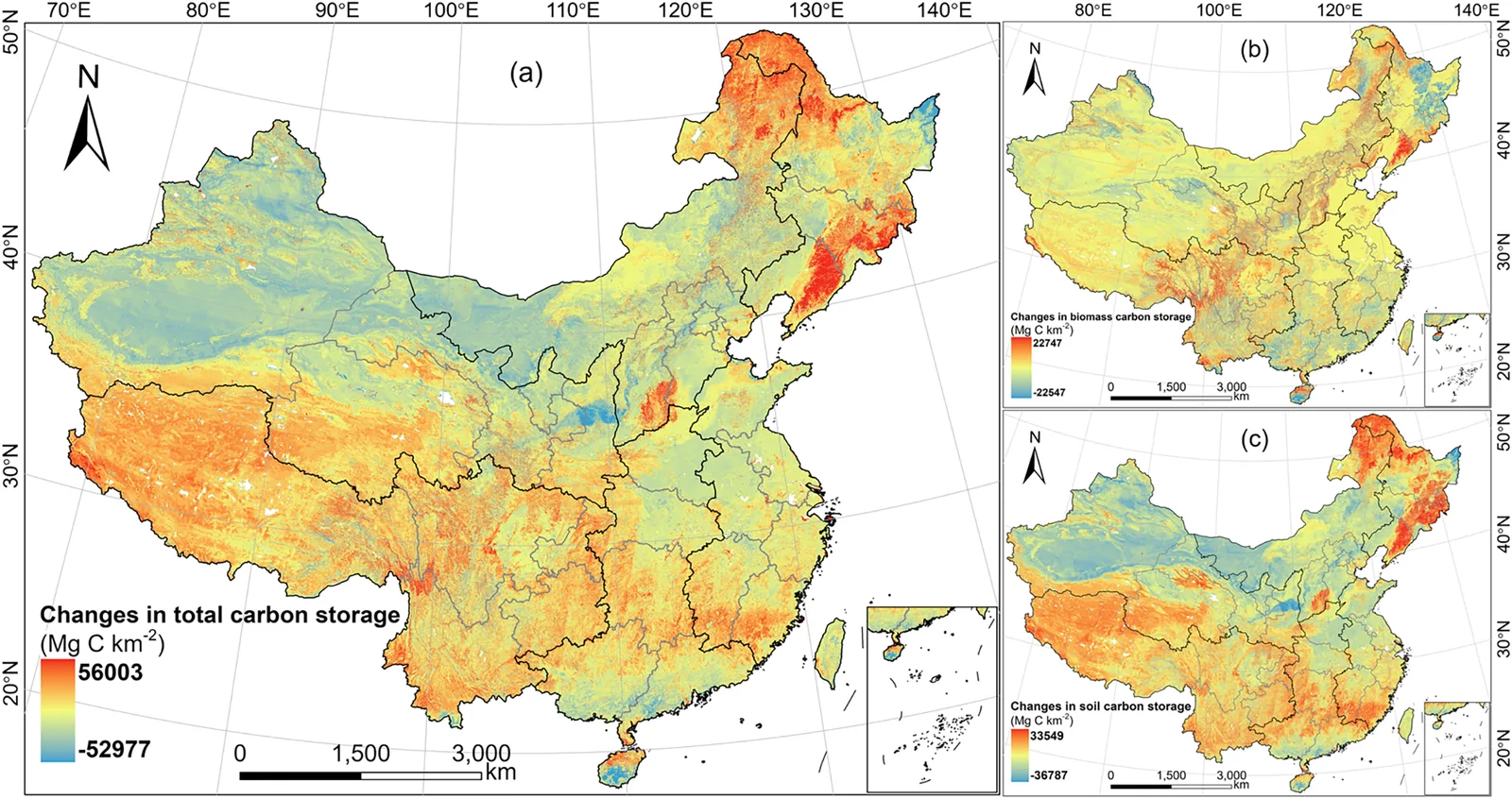
Spatial distribution of changes in 1-km grid-cell terrestrial carbon stock from 2014 to 2023. Panels show changes in (a) total carbon stock, (b) vegetation biomass carbon stock, and (c) soil organic carbon stock. Values are expressed as Mg C km^–2^. Map lines delineate the study area and do not necessarily represent accepted national boundaries.

Regional trajectories differed across time. From 1990 to 2002, area-normalized terrestrial carbon stock declined in all six regions, with the largest decreases in Midsouth China (−1.90 kg C m^–2^) and East China (−1.88 kg C m^–2^). These declines mainly reflected decreases in area-normalized vegetation biomass carbon stock. Forest vegetation biomass carbon accounted for most of these declines, decreasing by 1.69 and 1.45 kg C m^–2^ in Midsouth and East China, respectively.

From 2002 to 2012, area-normalized terrestrial carbon stock increased in five regions, while Southwest China showed a slight decline (−0.03 kg C m^–2^). Northeast China had the largest gain (+1.2 kg C m^–2^), reflecting an increase in area-normalized soil organic carbon stock (+1.73 kg C m^–2^) despite a concurrent decrease in area-normalized vegetation biomass carbon stock (−0.52 kg C m^–2^). Forest and cropland soils accounted for most of the soil carbon increase in this region, with gains of +0.86 and +0.84 kg C m^–2^, respectively.

After a short nationwide decline from 2012 to 2014, carbon stocks increased again from 2014 to 2023. During this period, area-normalized terrestrial carbon stock increased in all regions except Northwest China, where it declined by 0.22 kg C m^–2^. The largest gains occurred in Southwest China (+1.88 kg C m^–2^) and Northeast China (+1.70 kg C m^–2^), mainly reflecting increases in area-normalized soil organic carbon (+1.81 and +1.80 kg C m^–2^, respectively). These increases were concentrated in grassland soils in Southwest China (+1.05 kg C m^–2^) and forest soils in Northeast China (+1.32 kg C m^–2^). Midsouth, East, and North China also showed smaller gains of 0.76, 0.69, and 0.11 kg C m^–2^, respectively (Figures S3–S5).

The practical bootstrap analysis estimated the 2023 national terrestrial carbon stock was 113.48 Pg C, with a bootstrap interval of 109.15–134.14 Pg C (Table S5). The 2014–2023 national carbon-stock increase was 6.62 Pg C, with a bootstrap interval of 6.50–7.01 Pg C. Among regions, Southwest China had the largest carbon-stock gain during 2014–2023 (+4.40 Pg C; bootstrap interval: 4.34–4.50 Pg C), whereas Northwest China was the only region with a net carbon-stock loss (−0.65 Pg C; bootstrap interval: – 0.70 to – 0.34 Pg C).

### 3.4 Carbon-Stock Patterns Along Climatic Gradients

MAT and MAP were used to summarize broad climatic gradients across China. During 1990–2023, China’s MAT increased by 0.925 °C, whereas MAP decreased by 32.17 mm. Reconstructed carbon density varied markedly across the two-dimensional MAT-MAP space (Figures 8,S6). Higher total carbon density, vegetation biomass carbon density, and soil organic carbon density occurred more frequently in wetter climatic zones, whereas lower values were concentrated in dry regions with sparse vegetation cover. The MAT pattern was less monotonic. Vegetation biomass carbon density was generally higher under warm and humid conditions, while soil organic carbon density showed a more heterogeneous distribution along the temperature gradient. These patterns indicate that carbon density varies jointly with temperature and precipitation, but they should be interpreted as descriptive climatic-gradient patterns rather than evidence of independent or causal MAT/MAP effects.^50^

**Figure 8:**
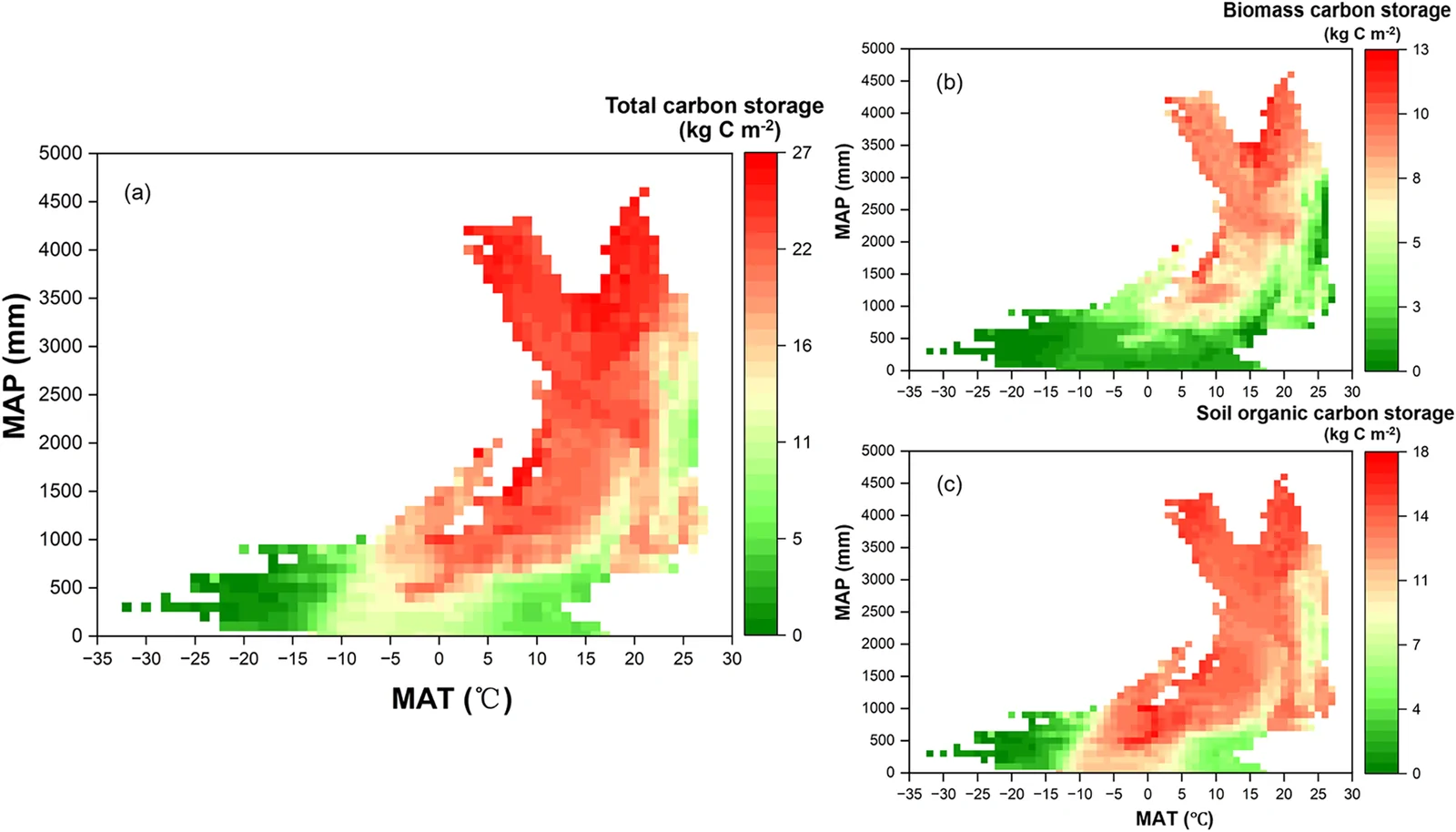
Area-normalized carbon stock along MAT and MAP gradients in China’s terrestrial ecosystems. Each point represents the area-normalized carbon stock within a 100 mm MAP bin and a 1 °C MAT bin. Panels show (a) total ecosystem carbon stock, (b) vegetation biomass carbon stock, and (c) soil organic carbon stock.

### 3.5 Carbon-Stock Changes in Major National Ecological Restoration Regions

From 2014 to 2023, mean ecosystem carbon density increased across all major ecological restoration project regions, with gains ranging from 0.47 to 1.74 kg C m^–2^ (Table 1). The Ecological Conservation and Restoration Project in the Three-River Source Region (ECRP) showed the largest increase, at 1.74 kg C m^–2^. This was followed by the Southwest China Karst Rocky Desertification Control Project (KRDCP, 1.41 kg C m^–2^), the Yangtze River Shelter Forest Project (YRSFP, 1.28 kg C m^–2^), the Zhujiang River Shelter Forest Project (ZRSFP, 1.01 kg C m^–2^), and the Natural Forest Protection Project (NFPP, 0.77 kg C m^–2^). Lower increases were observed in the Returning Grazing Land to Grassland Project (RGLGP, 0.65 kg C m^–2^) and the Grain for Green Program (GGP, 0.47 kg C m^–2^).

**Table 1:** Carbon-Stock Changes and Baseline-Adjusted Project-Region Gains in Major National Ecological Restoration Regions from 2014 to 2023^a^

project	carbon density in 2014 (kg C m^–2^)	carbon density in 2023 (kg C m^–2^)	gross carbon-stock change, 2014–2023 (Pg C)	gross annualized carbon-stock change (Pg C yr^–1^)	baseline-adjusted project-region carbon-stock gain, 2014–2023 (Pg C)	baseline-adjusted annualized gain (Pg C yr^–1^)
ECRP	11.3	13.04	0.683	0.068	0.049	0.005
NFPP	12.74	13.51	2.806	0.281	0.143	0.014
GGP	11.46	11.93	3.539	0.354	0.062	0.006
RGLGP	10.33	10.98	2.563	0.256	0.113	0.011
KRDCP	15.35	16.76	1.508	0.151	0.044	0.004
ZRSFP	15.63	16.64	0.428	0.043	0.031	0.003
YRSFP	13.14	14.42	3.230	0.323	0.052	0.005
Total	11.24	11.94	6.292	0.629	0.495	0.050

a Note. Values represent carbon-stock changes within ecological restoration project regions during 2014–2023. The Total row was calculated from the union of all seven project-region footprints, with overlapping areas counted only once. Project-specific rows are therefore not additive because some project regions overlap spatially. To avoid ambiguity, annualized values are calculated as average annual rates over the 2014–2023 map interval.

Across the seven project regions, total ecosystem carbon stock increased by 6.292 Pg C during 2014–2023, equivalent to an annualized increase of 0.629 Pg C yr^–1^ (Table 1). The GGP and YRSFP showed the largest gross annualized carbon-stock increases within project-region footprints, at 0.354 and 0.323 Pg C yr^–1^, respectively, whereas the ZRSFP showed the smallest increase, at 0.043 Pg C yr^–1^. In addition to gross changes within project regions, the baseline-adjusted project-region carbon-stock gain was estimated at 0.495 Pg C, equivalent to 0.050 Pg C yr^–1^. Among the seven projects, the NFPP had the largest baseline-adjusted project-region carbon-stock gain (0.143), followed by the RGLGP, GGP, YRSFP, ECRP, KRDCP, and ZRSFP. These estimates are presented as project-region accounting results rather than formal causal estimates of restoration effects, because project areas may differ from nonproject areas in climate, topography, soils, land-cover history, and initial ecosystem condition.

The ecological project-region sensitivity analysis showed that total carbon stock increased by 6.292 Pg C from 2014 to 2023 within the union of all project regions (Table S6). When carbon-stock changes were recalculated using a local three-year reference window centered on 2014, total carbon-stock change remained positive across all reference years, ranging from 4.082 to 6.292 Pg C, with a median of 6.146 Pg C. Soil organic carbon was the main contributor to the project-region carbon-stock increase, with a main 2014–2023 gain of 7.351 Pg C. These results indicate that the direction of project-region carbon-stock change was stable within the local reference window, although the magnitude varied. Therefore, the restoration-region results should be interpreted as scenario-based project-region accounting estimates rather than formal causal effects.

## 4 Discussion

### 4.1 Comparison with Previous National Carbon-Stock Estimates

Our long-term reconstruction demonstrates that China’s terrestrial ecosystems showed a substantial net carbon stock increase over the reconstruction period. The total carbon storage of approximately 113.48 Pg C falls within the range of previous national estimates. It closely aligns with the recent estimate by Yu et al.^18^ (111.58 Pg C), exceeds the estimates by Xu et al.^4^ (99.15 Pg C) and Tang et al.^2^ (89.27 Pg C), but remains lower than the estimate reported by Ni^51^ (154.99 Pg C) and Peng and Apps^21^ (157.9 Pg C).

These differences mainly reflect variation in data sources, reference years, spatial resolution, estimation methods, ecosystem coverage, and carbon-pool boundaries. Earlier studies based on coarse vegetation-type carbon-density methods, potential vegetation reconstructions, or empirical biosphere models^21,51^ used broader spatial units and different assumptions about ecosystem distribution and carbon density. Field-survey and synthesis-based studies^2,4^ are more directly constrained by observations, but differ in sampling period, ecosystem coverage, soil depth, and whether specific pools such as belowground biomass, litter, cropland biomass, wetlands, or barren lands are included. Our reconstruction integrates multisource field observations, annual land-cover information, and gridded environmental predictors across six major ecosystem types from 1990 to 2023. Therefore, differences among national estimates should be interpreted in relation to accounting boundaries, reference years, and upscaling methods, rather than as direct discrepancies among studies.

Forests remained the largest contributor to national terrestrial carbon stock in our reconstruction. In this study, forest carbon storage (47.99 Pg) accounted for 42.29% of the total carbon storage in 2023, aligning closely with previous estimates of approximately 45%.^52^ The estimated forest vegetation biomass carbon density was 66.6 Mg C ha^–1^, higher than several previous estimates for China, including 55.7 Mg C ha^–1^,^2^ 58.6 Mg C ha^–1^,^4^ 41.0 Mg C ha^–1^,^31^ 38.4 Mg C ha^–1^.^53^ This higher estimate may reflect both methodological differences and recent ecological changes, including forest recovery, forest maturation, and large-scale restoration programs. Because many of China’s forests are still young or middle-aged,^54^ continued stand development may provide additional biomass carbon-storage potential in the coming decades.^2,54,55^

### 4.2 Spatiotemporal Dynamics and Land-Use-Related Carbon-Stock Changes

The 1-km annual reconstruction reveals sustained but spatially heterogeneous carbon-stock changes across China from 1990 to 2023. Soil organic carbon dominated the national terrestrial carbon pool, storing approximately 3.9 times more carbon than vegetation biomass. This is consistent with previous studies showing that soil carbon accounts for the majority of terrestrial ecosystem carbon storage in China.^2,4^ Spatially, high vegetation carbon densities were concentrated mainly in humid and forested regions, whereas high soil organic carbon densities occurred in both humid forest regions and cold regions where decomposition is relatively slow.

Regional carbon-stock gains were closely associated with vegetation recovery and land-cover transitions. Southwest China accounted for 30.3% of the national terrestrial carbon stock in 2023, reflecting its extensive forest cover, humid climate, and high vegetation and soil carbon densities. This pattern is consistent with previous studies identifying Southwest China as an important region for soil and ecosystem carbon storage.^7^ Forest expansion, vegetation restoration, and ecological protection policies may have contributed to carbon-stock gains in southern and southwestern China,^56–58^ providing a plausible land-cover-related explanation for the observed regional patterns. In the Midsouth region, vegetation carbon density reached 3.76 kg C m^–2^, the highest among the six regions, further highlighting the importance of forested landscapes in regional vegetation carbon accumulation.^59,60^

Land-cover change also helps explain regional carbon losses. In Northeast China, vegetation biomass carbon declined by 0.10 Pg C over the past decade, which may be related to forest conversion or degradation. This interpretation is consistent with previous evidence that land-cover change caused substantial carbon losses in the region during earlier periods.^61^ In Northwest China, total carbon stock decreased by 0.65 Pg C, making it the only region with a net carbon-stock loss during the past decade. This decline was strongly associated with grassland loss:^62^ grassland area decreased by approximately 18,919 km^2^, accompanied by a 0.23 Pg C reduction in grassland vegetation biomass carbon. This grassland vegetation carbon loss accounted for approximately 35% of the total regional carbon-stock decline, indicating that grassland degradation or conversion was an important contributor to the regional carbon deficit.

These results suggest that land-cover transitions provide an important link between the observed spatial pattern of carbon-stock gains and losses. Conversion or degradation of forest and grassland can reduce vegetation biomass carbon rapidly, whereas soil carbon responses may occur more slowly and depend on disturbance intensity, soil moisture, and management history. Previous studies have shown that deforestation, grassland-to-cropland conversion, and overgrazing can substantially reduce soil organic carbon.^63–65^ Nevertheless, the associations reported here should be interpreted as land-cover-related evidence rather than formal causal attribution. Future work should combine land-cover transition matrices, field observations, and counterfactual or process-based analyses to separate the effects of land-use change, ecological restoration, climate variability, and ecosystem management on regional carbon-stock dynamics.

### 4.3 Climatic Context of Regional Carbon-Stock Dynamics

The climatic gradients observed in this study are consistent with known controls on vegetation and soil carbon, but they should not be interpreted as direct climate attribution. Temperature and precipitation affect carbon stocks through interacting pathways that also depend on land cover, soil properties, topography, ecosystem history, and management.^50^

Water availability is a major constraint on carbon accumulation in China. Higher precipitation generally supports vegetation productivity, canopy development, litter inputs, and belowground biomass, whereas water limitation in arid and semiarid regions restricts plant growth and organic matter inputs.^14^ This helps explain the lower carbon densities observed across dry climatic zones and the broad southeast-to-northwest contrast in ecosystem carbon density.^66^

Temperature effects are more complex. Warming can enhance plant growth in temperature-limited regions by lengthening the growing season, but it can also accelerate microbial decomposition, soil respiration, and soil organic carbon mineralization.^35,67,68^ These opposing processes help explain why vegetation biomass carbon and soil organic carbon did not show identical patterns along the MAT gradient. The high soil organic carbon density in Northeast China is consistent with the suppressing effect of low temperature on decomposition.^69–71^ In 2023, Northeast China had the highest soil organic carbon density among the six regions, reaching 12.3 kg C m^–2^, and showed an increase of 1.80 kg C m^–2^ over the past decade.

Overall, the MAT-MAP results provide a descriptive climatic context for regional carbon-stock patterns, rather than a separation of climate, land-use, and management effects. Future work combining process-based modeling, long-term observations, and formal attribution analyses will be needed to quantify the relative contributions of climate change, land-use transitions, ecological restoration, and ecosystem management.^72^

### 4.4 Scenario-Based Accounting of Carbon-Stock Changes in Ecological Restoration Regions

Large-scale ecological restoration has been a central component of China’s land-management policy since the late 1970s. Previous studies have linked afforestation, reforestation, grazing exclusion, and ecological protection to vegetation recovery and carbon-stock increases in several regions of China.^7,25,73–75^ These studies provide useful ecological context for interpreting the carbon-stock gains observed within major restoration regions, particularly through biomass accumulation, reduced vegetation degradation, and gradual soil organic carbon recovery.^7,76^ China’s afforestation initiatives led to a 40% increase in forest biomass carbon storage between the 1970s and 2000s,^75^ and reforestation combined with evolving tree age structure has increased forest carbon storage by approximately 13%.^32^

Previous studies have linked national ecological restoration programs to carbon-stock increases in project areas.^49^ Recent research estimates that, during the first decade of the 21st century, several major ecological restoration projects in China contributed a cumulative carbon sink of 0.77 Pg C, with an average annual sequestration rate of 0.074 Pg C yr^–1^.^49^ In this study, total ecosystem carbon stock increased by 6.292 Pg C within the seven major ecological restoration regions during 2014–2023. Using the baseline-adjusted accounting approach, the baseline-adjusted project-region carbon-stock gain was estimated at 0.495 Pg C, equivalent to 0.050 Pg C yr^–1^. Among the project regions, the NFPP showed the largest baseline-adjusted gain, followed by the RGLGP, the GGP, the YRSFP, and the ECRP. These patterns are broadly consistent with the expected effects of forest protection, vegetation recovery, grazing reduction, and soil-conservation measures on ecosystem carbon accumulation.^49,77^

These estimates should be interpreted as approximate project-region accounting results. Restoration areas were not randomly assigned and may differ from nonproject areas in climate, topography, soil properties, land-cover history, degradation status, and initial carbon density. Therefore, average carbon-density changes in nonproject areas may not fully represent the counterfactual baseline that would have occurred within project areas in the absence of restoration. The project-region results nevertheless provide a useful national-scale accounting of carbon-stock changes within major restoration areas. They suggest that these regions generally experienced carbon-stock gains over the past decade, but the magnitude of the estimated gain depends on project extent, ecosystem type, initial land-cover condition, and the assumed baseline. Future work should strengthen attribution to separate restoration effects from background climate variability, land-cover transitions, and broader ecosystem management.

## Supplementary Material



## Data Availability

The annual carbon-density raster data set has been deposited in Zenodo at 10.5281/zenodo.21372793. The data set is organized into four archives, AGBC.zip, BGBC.zip, SOC20.zip, and SOC100.zip, each containing annual 1-km GeoTIFF files from 1990 to 2023. The monthly temperature and precipitation data used to derive annual MAT and MAP are available from the National Earth System Science Data Center of China (https://www.geodata.cn). The annual China Land Cover Data set is available from Zenodo at 10.5281/zenodo.15853565. The field carbon-density data set for Chinese terrestrial ecosystems in the 2010s was obtained from the National Ecosystem Science Data Center of China (https://www.nesdc.org.cn).

## Notes

Additional validation metrics and uncertainty and sensitivity analyses; temporal changes in vegetation biomass carbon, soil organic carbon, and total carbon stock across ecosystem types and regions in China from 1990 to 2023; spatial distributions of carbon-stock changes from 2014 to 2023; reconstructed carbon density along mean annual precipitation and temperature gradients; spatial extent and implementation periods of the seven major national ecological restoration projects; ecosystem-specific carbon stocks and densities in 2023; and comparison with previously reported national terrestrial carbon-stock estimates for China (PDF)
