# Supplementary material for: High-Resolution Assessment of Terrestrial Carbon Storage and Restoration Benefits in China, 1990–2023
Source: Environ Sci Technol. 2026 Aug 31;60(36):25371–84. doi: 10.1021/acs.est.6c00827 (PMC13585055; doi:10.1021/acs.est.6c00827)
Supplement: Supplementary file 1 [file es6c00827_si_001.pdf]

Supporting Information

High-Resolution Assessment of Terrestrial Carbon Storage and Restoration Benefits in China, 1990-2023

Zhen Wu,[a,b,c] Xianjin Huang,[a,b]* Michael E. Meadows [a,d]

[a] School of Geography and Ocean Science, Nanjing University, Nanjing, Jiangsu 210023, China

[b] The Key Laboratory of Carbon Neutrality and Territory Optimization, Ministry of Natural Resources, Nanjing, Jiangsu 210023, China

[c] Department of Geography and Resource Management, The Chinese University of Hong Kong, Shatin, Hong Kong SAR, China

[d] Department of Environmental & Geographical Science, University of Cape Town, Rondebosch 7701, South Africa

***Corresponding author:** Xianjin Huang (hxj369@nju.edu.cn)

<u>This file includes:</u>

Supplementary Methods

Supplementary Figures S1-S7

Supplementary Tables S1-S9

Supplementary Methods | Additional validation metrics.
Figure S1 | Temporal changes in vegetation biomass carbon stock, soil organic carbon stock, and total carbon stock in China’s terrestrial ecosystems from 1990 to 2023.
Figure S2 | Temporal changes in terrestrial carbon stock by ecosystem type and region from 1990 to 2023.
Figure S3 | Spatial distribution of changes in 1-km grid-cell total carbon stock in China’s terrestrial ecosystems from 2014 to 2023.
Figure S4 | Spatial distribution of changes in 1-km grid-cell vegetation biomass carbon stock in China’s terrestrial ecosystems from 2014 to 2023.
Figure S5 | Spatial distribution of changes in 1-km grid-cell soil organic carbon stock in China’s terrestrial ecosystems from 2014 to 2023.
Figure S6 | Area-normalized carbon stocks along MAP and MAT gradients in China’s terrestrial ecosystems.
Figure S7 | Spatial distribution of the seven major national ecological restoration project regions.
Table S1 | Overall independent validation and carbon-pool-specific external validation metrics.
Table S2 | Site-ID-cluster bootstrap intervals for overall validation metrics.
Table S3 | Six-region validation diagnostics.
Table S4 | Temporal-period external validation metrics.
Table S5 | Practical bootstrap uncertainty intervals for national and regional terrestrial carbon-stock estimates.
Table S6 | Local reference-year sensitivity of carbon-stock changes within the union of ecological restoration project regions.
Table S7 | Ecosystem-specific vegetation biomass carbon stocks, soil organic carbon stocks, and carbon densities in China in 2023.
Table S8 | Comparison of national terrestrial carbon-stock estimates for China reported in previous studies.
Table S9 | Spatial extent and implementation period of the seven major national ecological restoration projects in China.

Supplementary Methods | Additional validation metrics

Let y_i and $\hat{y}_i$ denote the observed and predicted carbon density, respectively, for validation record i, and let $\grave{y}$ denote the mean observed carbon density across n validation records.

1. Coefficient of determination (R²)

The coefficient of determination was calculated from the residual sum of squares (SSE) and total sum of squares (SST) as follows:

$$R^2 = 1 - \frac{SSE}{SST} (S1)$$

Where, $SSE = \sum_{i=1}^{n} (y_i - \hat{y}_i)^2 (S2)$

$$SST = \sum_{i=1}^{n} (y_i - \grave{y})^2 (S3)$$

Thus,

$$R^2 = 1 - \frac{\sum_{i=1}^{n} (y_i - \hat{y}_i)^2}{\sum_{i=1}^{n} (y_i - \grave{y})^2} (S4)$$

2. Squared Pearson correlation (r²)

The Pearson correlation coefficient between observed and predicted values was calculated as:

$$r = \frac{\sum_{i=1}^{n} (y_i - \grave{y})(\hat{y}_i - \grave{\hat{y}})}{\sqrt{\sum_{i=1}^{n} (y_i - \grave{y})^2} \sqrt{\sum_{i=1}^{n} (\hat{y}_i - \grave{\hat{y}})^2}} (S5)$$

The squared Pearson correlation was then calculated as:

$$r^2 = \left[\frac{\sum_{i=1}^{n} (y_i - \grave{y})(\hat{y}_i - \grave{\hat{y}})}{\sqrt{\sum_{i=1}^{n} (y_i - \grave{y})^2} \sqrt{\sum_{i=1}^{n} (\hat{y}_i - \grave{\hat{y}})^2}} \right]^2 (S6)$$

3. Mean absolute error (MAE)

MAE was calculated as:

$$MAE = \frac{1}{n}\sum_{i=1}^{n} | y_i - \hat{y}_i | (S7)$$

4. Root mean square error (RMSE)

RMSE was calculated as:

$$RMSE = \sqrt{\frac{1}{n}\sum_{i=1}^{n} (y_i - \hat{y}_i)^2 (S8)}$$

5. Mean bias error (Bias)

Mean bias error was calculated as:

$$MBE = \frac{1}{n}\sum_{i=1}^{n} (\hat{y}_i - y_i) \qquad \text{(S9)}$$

where positive values indicate overestimation by the model on average, and negative values indicate underestimation. MBE is reported in units of kg C m^{-2}.

6. Lin's concordance correlation coefficient (CCC)

Lin's concordance correlation coefficient was used to evaluate the agreement between observed and predicted values by jointly considering correlation and deviation from the 1:1 line. It was calculated as:

$$CCC = \frac{2\rho\sigma_y\sigma_{\hat{y}}}{\sigma_y^2 + \sigma_{\hat{y}}^2 + (\grave{y} - \grave{\hat{y}})^2} (S10)$$

Where, ρ is the Pearson correlation coefficient between y_i and $\hat{y}_i$, σ_y^2 and $\sigma_{\hat{y}}^2$ are the variances of the observed and predicted values, respectively. Equivalently, CCC can be expressed using the covariance between observed and predicted values:

$$CCC = \frac{2\sigma_{y\hat{y}}}{\sigma_y^2 + \sigma_{\hat{y}}^2 + (\grave{y} - \grave{\hat{y}})^2} (S11)$$

Where $\sigma_{y\hat{y}}$ denotes the covariance between observed and predicted values.

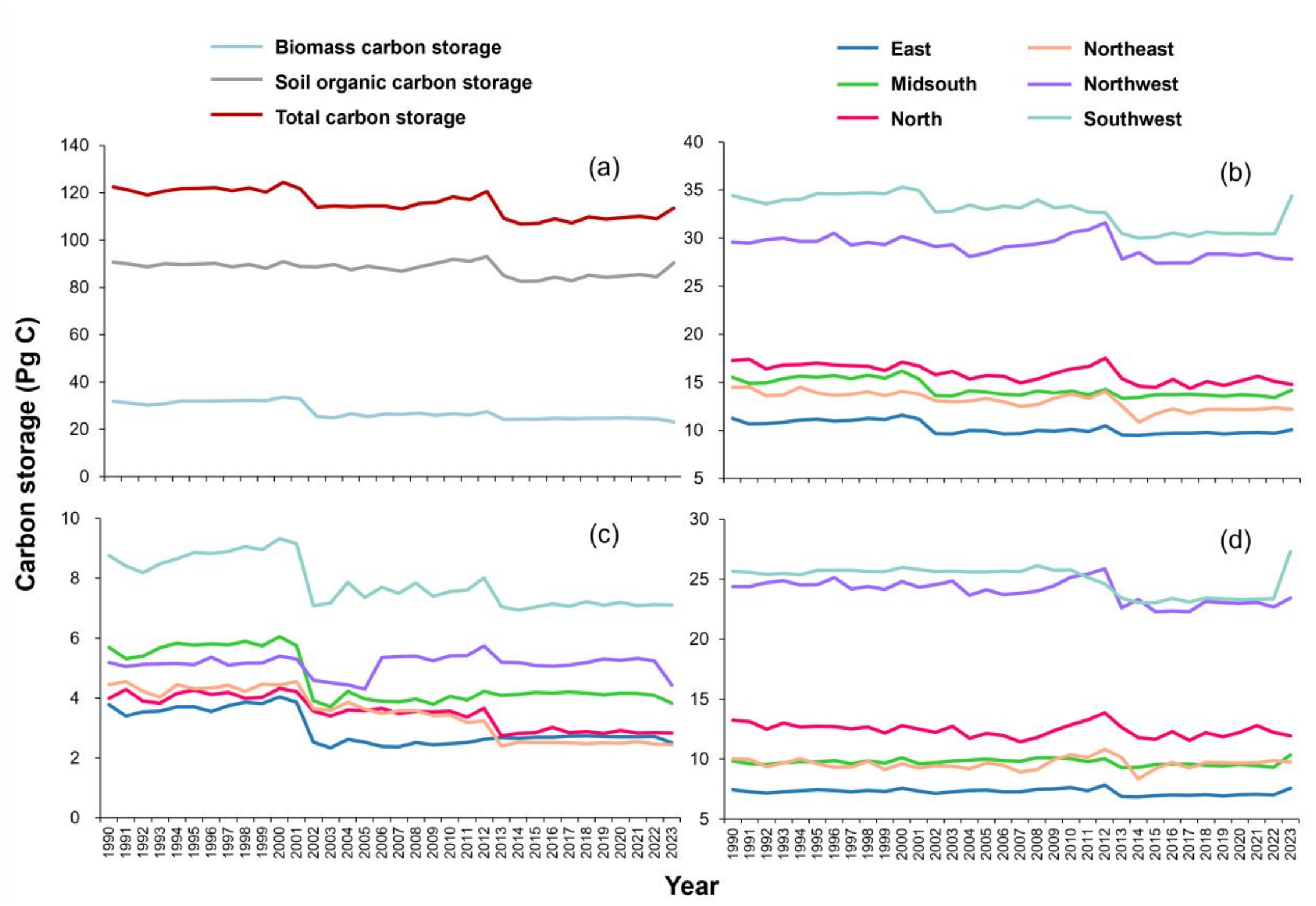

Figure S1 | Temporal changes in vegetation biomass carbon stock, soil organic carbon stock, and total carbon stock in China's terrestrial ecosystems from 1990 to 2023. Panels show (a) vegetation biomass, soil organic carbon, and total carbon stocks in China; (b) total carbon stocks by region; (c) vegetation biomass carbon stocks by region; and (d) soil organic carbon stocks by region.

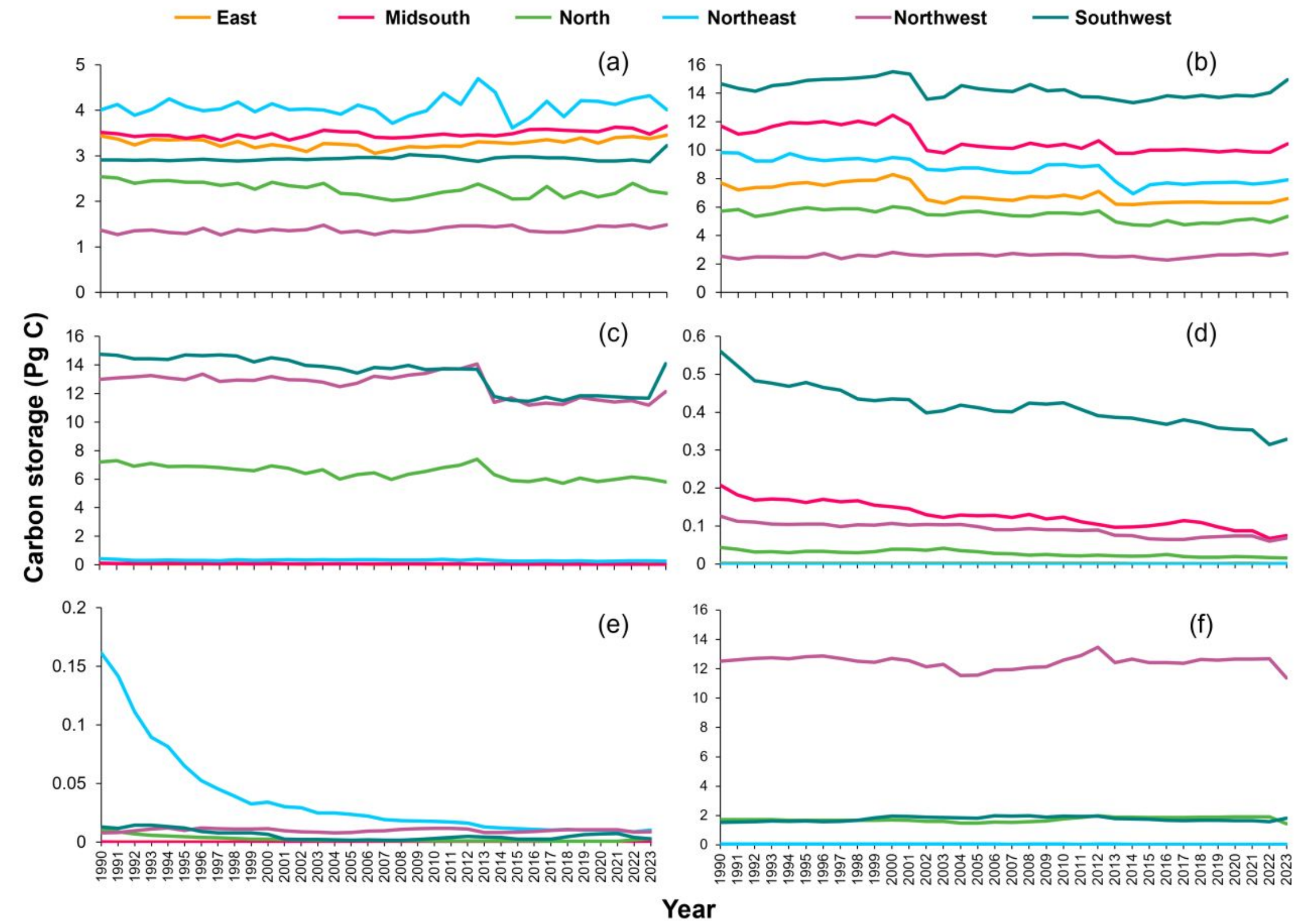

Figure S2 | Temporal changes in terrestrial carbon stock by ecosystem type and region from 1990 to 2023. Panels show (a) croplands, (b) forests, (c) grasslands, (d) shrublands, (e) wetlands, and (f) barren lands.

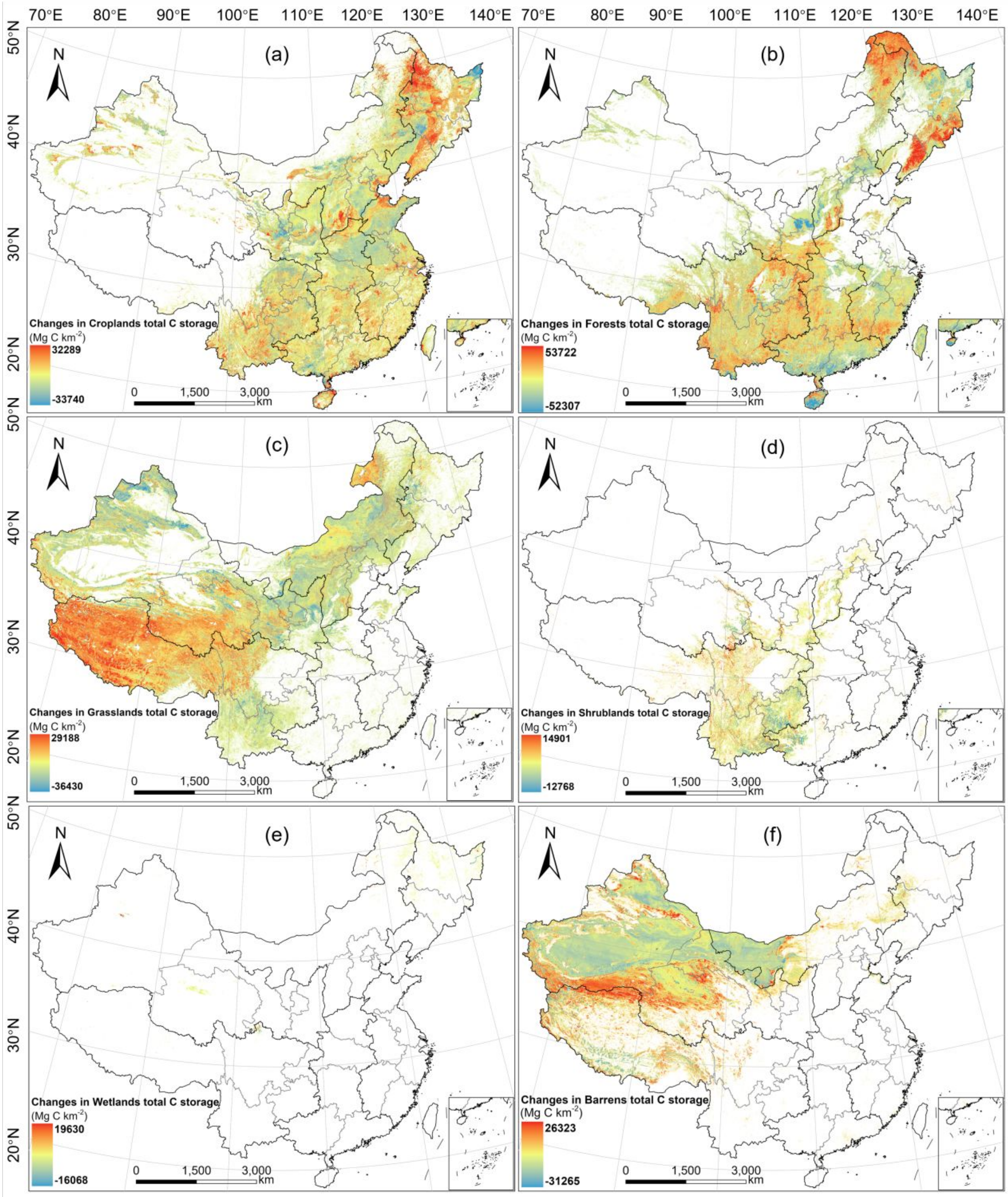

Figure S3 | Spatial distribution of changes in 1-km grid-cell total carbon stock in China's terrestrial ecosystems from 2014 to 2023. Panels show (a) croplands, (b) forests, (c) grasslands, (d) shrublands, (e) wetlands, and (f) barren lands. Values are expressed as Mg C km^{-2}. Map lines delineate study areas and do not necessarily depict accepted national boundaries.

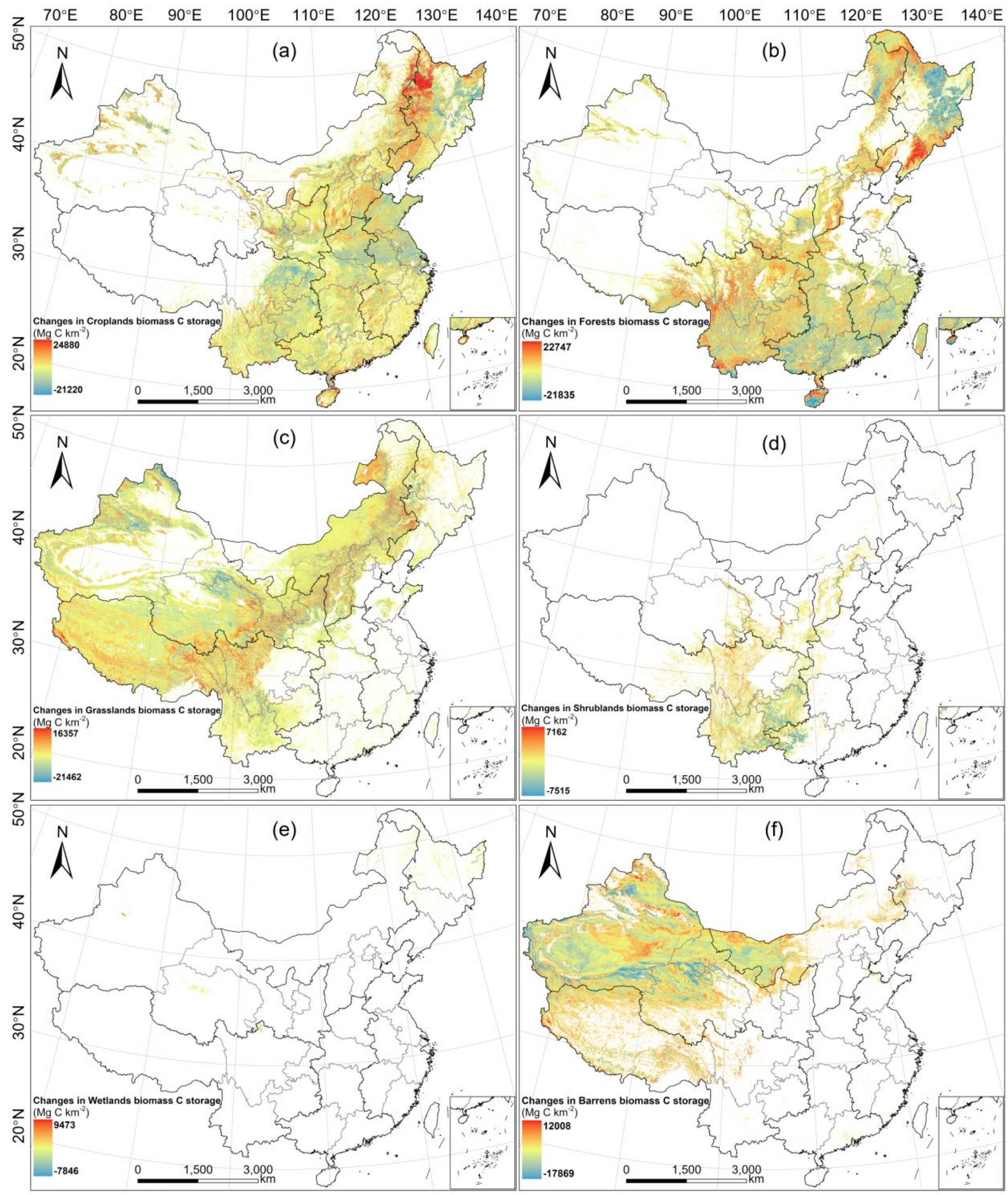

Figure S4 | Spatial distribution of changes in 1-km grid-cell vegetation biomass carbon stock in China's terrestrial ecosystems from 2014 to 2023. Panels show (a) croplands, (b) forests, (c) grasslands, (d) shrublands, (e) wetlands, and (f) barren lands. Values are expressed as Mg C km^{-2}. Map lines delineate study areas and do not necessarily depict accepted national boundaries.

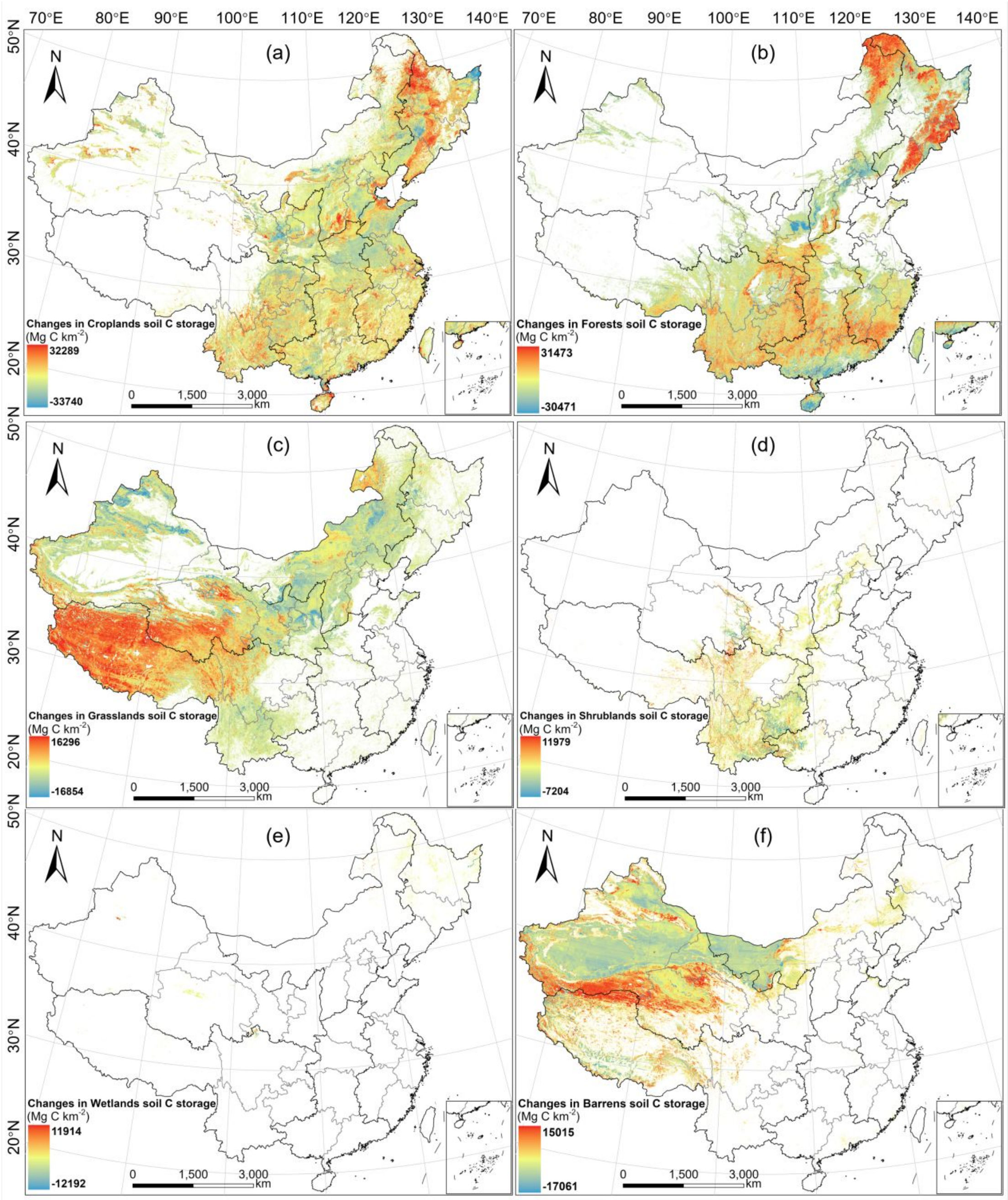

Figure S5 | Spatial distribution of changes in 1-km grid-cell soil organic carbon stock in China's terrestrial ecosystems from 2014 to 2023. Panels show (a) croplands, (b) forests, (c) grasslands, (d) shrublands, (e) wetlands, and (f) barren lands. Values are expressed as Mg C km^{-2}. Map lines delineate study areas and do not necessarily depict accepted national boundaries.

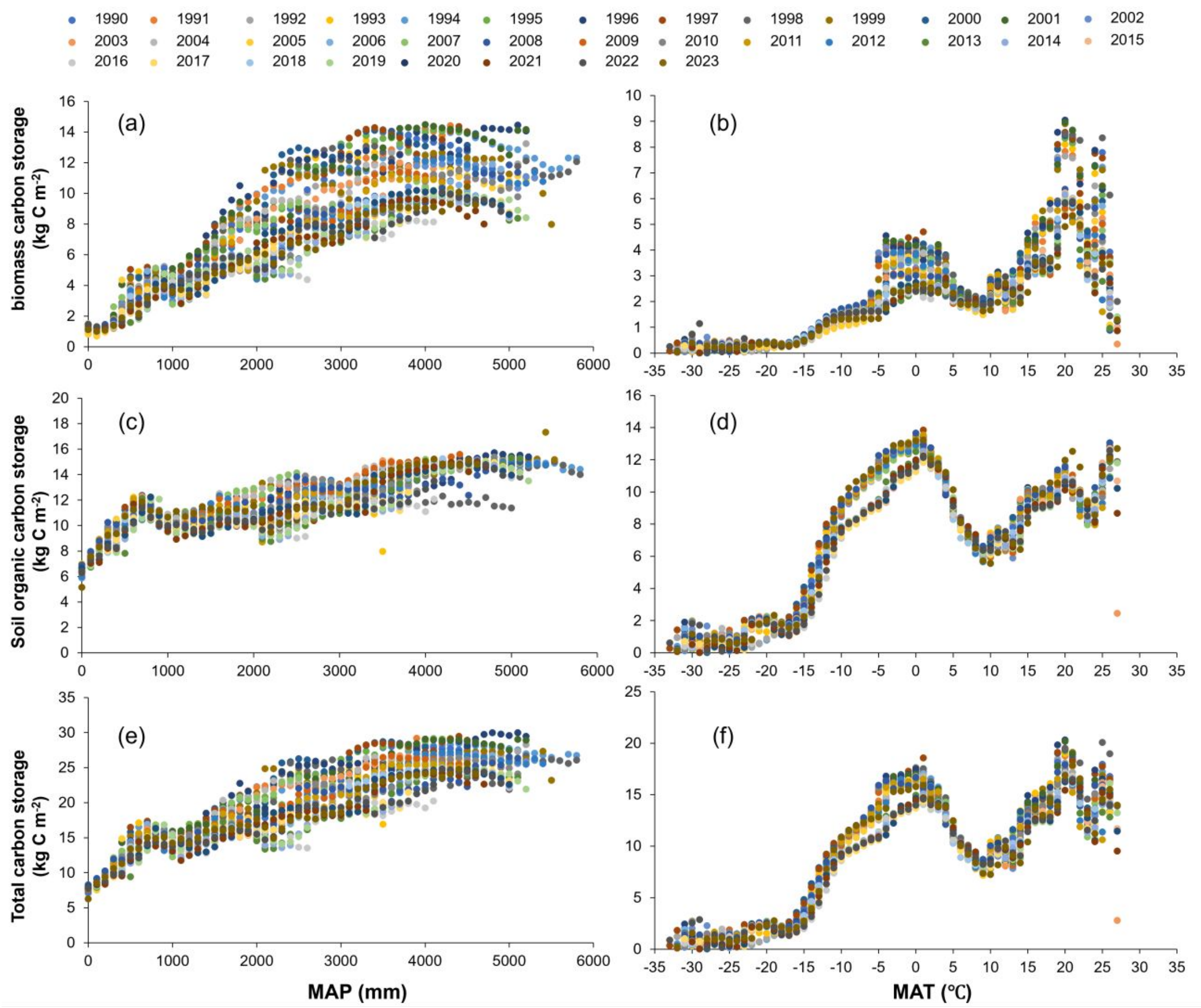

Figure S6 | Area-normalized carbon stocks along MAP and MAT gradients in China's terrestrial ecosystems. Panels (a) and (b) show area-normalized vegetation biomass carbon stock along MAP and MAT gradients, respectively; panels (c) and (d) show area-normalized soil organic carbon stock; and panels (e) and (f) show total area-normalized carbon stock. Values are expressed as kg C m^{-2}.

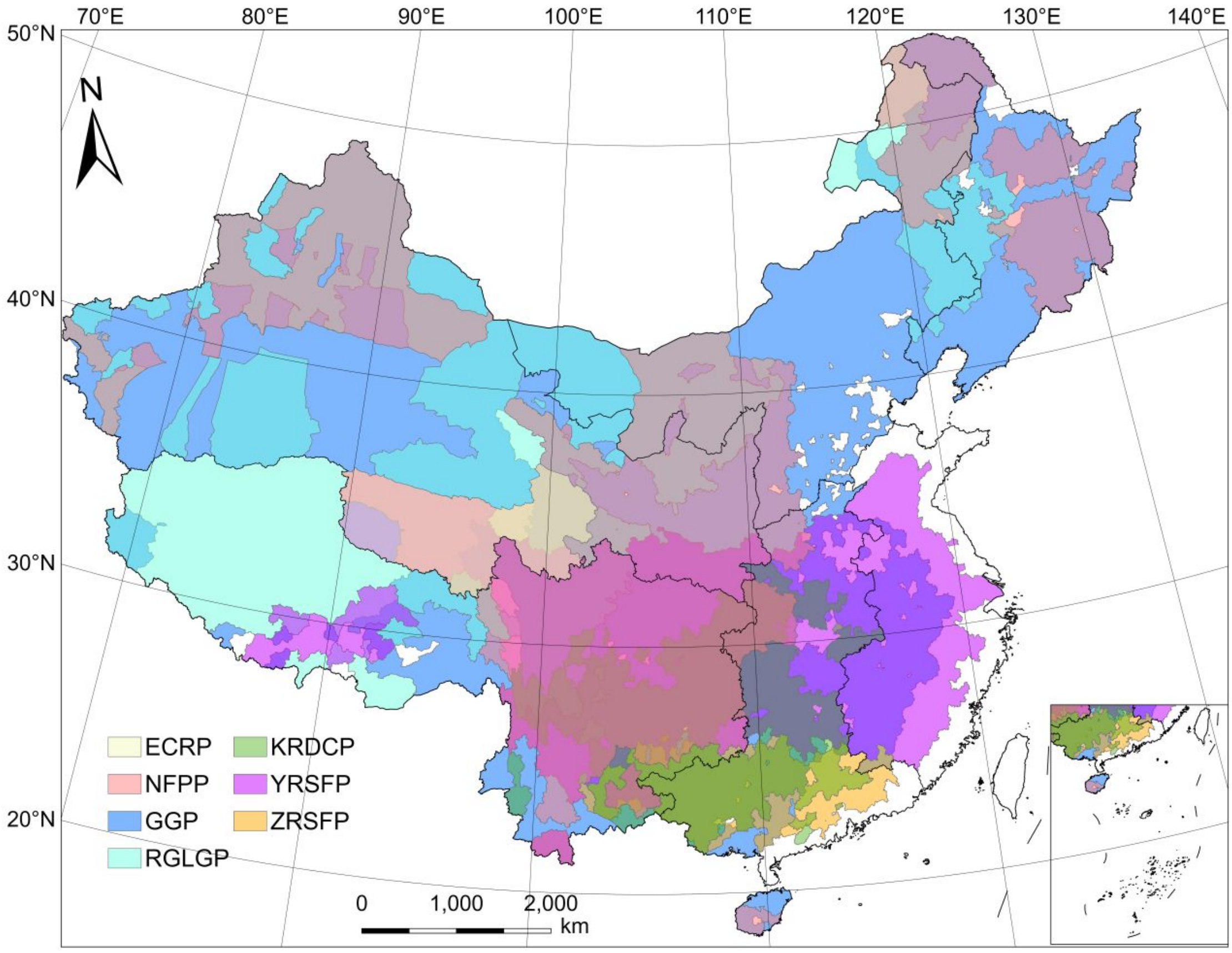

Figure S7 | Spatial distribution of the seven major national ecological restoration project regions. Map lines delineate the study area and do not necessarily depict accepted national boundaries.

Table S1 | Overall independent validation and carbon-pool-specific external validation metrics.

Analysis	n	Unique sites	R^2	Pearson r^2	MAE	RMSE	Bias	CCC
All records	1148	196	0.698	0.720	1.506	2.442	0.120	0.797
Aggregated by PID-year-pool	783	166	0.693	0.713	1.704	2.790	-0.058	0.794
AGBC	178	87	0.520	0.643	2.544	3.396	-0.930	0.628
BGBC	78	76	-0.364	0.203	0.892	1.155	0.510	0.398
SOC100	199	41	0.563	0.581	2.728	4.125	-0.107	0.688
SOC20	693	52	0.595	0.758	0.957	1.372	0.411	0.688

Note. Sites represent unique spatial site IDs. R^2 is calculated as 1 − SSE/SST. Bias is predicted minus observed. Units for MAE, RMSE, and Bias are kg C m^{-2}. CCC is Lin's concordance correlation coefficient. BGBC is retained in the main validation set and interpreted cautiously because of weaker external agreement.

Table S2 | Site-ID-cluster bootstrap intervals for overall validation metrics.

Metric	Point estimate	Bootstrap 2.5%	Bootstrap 97.5%
R^2	0.698	0.637	0.741
Pearson r^2	0.720	0.651	0.799
MAE	1.506	1.212	1.881
RMSE	2.442	1.870	3.088
Bias	0.120	-0.335	0.520
MedianAE	0.939	0.725	1.230
CCC	0.797	0.751	0.837

Note. Intervals were estimated using 1,000 site-ID-cluster bootstrap resamples. Units for MAE, RMSE, and Bias are kg C m^{-2}.

Table S3 | Six-region validation diagnostics.

Region	n	Unique sites	R^2	Pearson r^2	MAE	RMSE	Bias	CCC
East	70	23	0.468	0.651	1.910	2.123	1.245	0.709
Midsouth	54	4	-1.019	0.270	3.366	4.271	-3.165	0.33
North	178	36	0.713	0.764	1.187	1.524	0.639	0.838
Northeast	22	1	-2.023	0.809	3.388	3.996	3.146	0.535
Northwest	765	122	0.711	0.795	1.144	1.915	0.181	0.789
Southwest	59	10	0.408	0.464	4.276	5.790	-1.704	0.585

Note. Regional statistics based on few unique site-IDs (Midsouth; Northeast) are reported as exploratory diagnostics and should not be interpreted as regionally representative map-accuracy estimates.

Table S4 | Temporal-period external validation metrics.

Analysis	n	Unique sites	R^2	Pearson r^2	MAE	RMSE	Bias	CCC
Calibration-overlap external validation (2003–2014)	793	178	0.705	0.726	1.628	2.592	0.177	0.804
Post-calibration external validation (2015–2020)	355	75	0.668	0.704	1.232	2.068	-0.009	0.766

Note. The post-calibration period partially evaluates predictions after the 2000–2014 calibration-supported window. It does not directly validate the 1990–1999 hindcast or 2021–2023 estimates.

Table S5 | Practical bootstrap uncertainty intervals for national and regional terrestrial carbon-stock estimates.

Region	2023 carbon stock (Pg C)	Bootstrap interval for 2023 stock (Pg C)	2014-2023 carbon-stock change (Pg C)	Bootstrap interval for 2014-2023 change (Pg C)
National	113.48	109.15-134.14	6.62	6.50-7.01
East	10.09	9.39-12.45	0.59	0.53-0.61
Midsouth	14.19	13.23-17.95	0.77	0.72-0.79
North	14.77	14.11-17.67	0.17	0.14-0.26
Northeast	12.21	11.52-14.67	1.35	1.32-1.36
Northwest	27.83	26.08-33.10	−0.65	−0.70 to −0.34
Southwest	34.38	32.89-40.19	4.40	4.34-4.50

Note. Bootstrap intervals represent the 2.5th and 97.5th percentiles from 1,000 site-ID-cluster residual-bootstrap iterations. Residuals were calculated from the independent validation dataset as predicted minus observed carbon density and were resampled as site-ID/spatial-ID clusters. In each iteration, bootstrapped residual corrections were applied to the reconstructed carbon densities within the corresponding carbon-pool and ecosystem strata where validation support was sufficient, and carbon stocks were recalculated by multiplying the adjusted carbon densities by ecosystem area. For strata with limited validation support, carbon-pool-level residual distributions were used as fallback residual pools. These intervals provide a practical external-validation-based uncertainty characterization and do not represent a full probabilistic propagation of all sources of uncertainty.

Table S6 | Local reference-year sensitivity of carbon-stock changes within the union of ecological restoration project regions.

Carbon component	Main 2014-2023 change (Pg C)	Median change using 2013-2015 reference window (Pg C)	Sensitivity range using 2013-2015 reference window (Pg C)	Direction consistency
Vegetation biomass carbon	−1.060	−1.060	−1.177 to −0.973	3/3 negative
Soil organic carbon	7.351	7.323	5.055 to 7.351	3/3 positive
Total carbon stock	6.292	6.146	4.082 to 6.292	3/3 positive

Note. The main estimate represents the 2014–2023 carbon-stock change. The local sensitivity analysis used a three-year reference window centered on 2014, including 2013, 2014, and 2015. Direction consistency indicates whether the sign of carbon-stock change was consistent across the three reference-year comparisons.

Table S7 | Ecosystem-specific vegetation biomass carbon stocks, soil organic carbon stocks, and carbon densities in China in 2023.

	Total carbon storage (Pg C)		Carbon density (kg C m^{-2})	
	**Biomass**	**Soil**	**Biomass**	**Soil**
Croplands	\	18.0	\	1.89
Forests	16.25	31.74	1.71	3.33
Grasslands	4.54	27.78	0.48	2.92
Shrublands	0.15	0.34	0.02	0.04
Wetlands	0.007	0.017	0.001	0.002
Barren lands	2.2	12.45	0.23	1.31
Total	23.15	90.33	2.43	9.48

Table S8 | Comparison of national terrestrial carbon-stock estimates for China reported in previous studies.

	Year	Vegetation biomass carbon storage (Pg C)	Soil organic carbon storage (Pg C)	Total carbon storage (Pg C)	References
Croplands	1979-1985	\	15.04	\	[1]
	1979-1985	\	15.1±3.56	\	[2]
	2000-2014	\	15.17±1.99	\	[2]
	2000	\	16.592	\	[3]
	2020	\	16.349	\	[3]
	1981-1998	0.98	18.73	19.71	[4]
	1980s-2010s	2.0	11.18	13.18	[5]
	2008	0.49	7.42	7.91	[6]
	2000-2014	\	15.17	15.17	[7]
	2011-2015	0.55	15.77	16.32	[8]
	1990-2023	\	18.0	18.0	This study
Forests	1981–1998	11.51	35.49	47	[4]
	1989-1993	6.2	21.02	28.116	[9]
	1980s-2010s	7.74	22.31	29.77	[5]
	2008	10.42	11.28	21.7	[6]
	2000-2014	11.49	22.59	34.08	[7]
	2011-2015	10.48	19.98	30.46	[8]
	1990-2023	16.25	31.74	47.99	This study
Grasslands	1981–1988	0.99	28.11	29.1	[10]
	1981–1988	3.06	41.03	44.09	[11]
	1981–1998	0.56	16.69	17.25	[4]
	1983-1993	4.7	53.7	58.4	[12]
	1980s-2010s	2.52	43.0	45.51	[5]
	1961—2013	3.2	56.3	59.5	[13]
	2008	3.96	12.93	16.89	[6]
	2000-2014	1.94	23.75	25.69	[7]
	2003–2014	1.61	29.37	30.98	[14]
	2011-2015	1.35	24.03	25.38	[8]
	1990-2023	4.54	27.78	32.32	This study
Shrublands	1981–1988	0.2	11.8	12	[11]
	1980s-2010s	0.85	9.1	9.95	[5]
	2000-2014	0.44	6.98	7.42	[7]
	2008	3.32	5.64	8.96	[6]
	2011-2015	0.71	5.91	6.62	[8]
	1990-2023	0.15	0.34	0.49	This study
Wetlands	1980s-2010s	0.24	4.57	4.81	[5]
	2000-2014	0.2	3.41	3.61	[7]

	1979-1985	\	1.75	\	[1]
	1979-1985	\	4.51±1.63	\	[2]
	2000-2014	\	3.75±0.89	\	[2]
	1999-2018	0.22	16.7	16.92	[15]
	2007-2009	0.27	6.18	6.45	[16]
	2005-2017	\	\	0.0746±0.004	[17]
	1990-2023	0.007	0.017	0.02	This study
Barren lands	1980s-2010s	0.47	7.89	8.36	[5]
	2000-2014	0.53	12.65	13.18	[7]
	1990-2023	2.2	12.45	14.65	This study
Terrestrial ecosystems	1979-1985	\	83.46±11.89	\	[2]
	2000-2014	\	86.5±8.71	\	[2]
	2000	\	86.074	\	[3]
	2020	\	86.771	\	[3]
	1970-1980s	6.1	185.7	191.8	[18]
	1983-1993	35.23	119.76	154.99	[12]
	1990s	57.9	100	157.9	[19]
	1981-1998	13.29	82.68	95.97	[4]
	Late 20 century	13.91	82.78	96.69	[20]
	2008	18.19	37.27	55.46	[6]
	1980s-2010	13.56	98.03	111.58	[5]
	2000-2014	14.6	84.55	99.15	[7]
	2011-2015	14.29	74.98	89.27	[8]
	1990-2023	23.15	90.33	113.48	This study

Table S9 | Spatial extent and implementation period of the seven major national ecological restoration projects in China.

National ecological restoration projects	Area (10^4 km^2)	Start year	Boundary source
Ecological Conservation and Restoration Project in Three-river Source Region (ECRP)	38.87	2005	[21]
Natural Forest Protection Project (NFPP)	360.01	2000	[22]
Grain for Green Program (GGP)	741.8	2002	[23]
Returning Grazing Land to Grassland Project (RGLGP)	389.03	2003	[24]
Southwest China Karst Rocky Desertification Control Project (KRDCP)	105.18	2008	[25]
Zhujiang River Shelter Forest Project (ZRSFP)	41.33	1996	[26]
Yangtze River Shelter Forest Project (YRSFP)	250.79	1989	[27]

References:

[1] Xu L, He N, Yu G, et al. Differences in pedotransfer functions of bulk density lead to high uncertainty in soil organic carbon estimation at regional scales: Evidence from chinese terrestrial ecosystems. Journal of Geophysical Research: Biogeosciences, 2015, 120: 1567-1575. https://doi.org/10.1002/2015JG002929

[2] Xu L, Yu G, He N. Increased soil organic carbon storage in chinese terrestrial ecosystems from the 1980s to the 2010s. Journal of Geographical Sciences, 2019, 29: 49-66. https://doi.org/10.1007/s11442-019-1583-4

[3] Tong R, Liang X, Guan Q, et al. Estimation of soil carbon storage change from land use and management at a high spatial resolution in china during 2000-2020. Acta Geographica Sinica, 2023, 78: 2209-2222. https://doi.org/10.11821/dlxb202309006

[4] Li K, Wang S, Cao M. Vegetation and soil carbon storage in china. Science in China Series D: Earth Sciences, 2004, 47: 49-57. https://doi.org/10.1360/02yd0029

[5] Yu G, Li X, Wang Q, et al. Carbon storage and its spatial pattern of terrestrial ecosystem in china. Journal of Resources and Ecology, 2010, 1: 97-109. https://doi.org/10.3969/j.issn.1674-764x.2010.02.001

[6] Wang LX, Gao JX, Shen WM, et al. Carbon storage in vegetation and soil in chinese ecosystems estimated by carbon transfer rate method. Ecosphere, 2021, 12: e03341. https://doi.org/10.1002/ecs2.3341

[7] Xu L, Yu GR, He NP, et al. Carbon storage in china's terrestrial ecosystems: A synthesis. Scientific Reports, 2018, 8: 2806. https://doi.org/10.1038/s41598-018-20764-9

[8] Tang X, Zhao X, Bai Y, et al. Carbon pools in china’s terrestrial ecosystems: New estimates based on an intensive field survey. Proceedings of the National Academy of Sciences, 2018, 115: 4021-4026. https://doi.org/10.1073/pnas.1700291115

[9] Zhou Y, Yu Z, Zhao S. Carbon storage and budget of major chinese forest types. Chinese Journal of Plant Ecology, 2000, 24: 518-522.

[10] Fang J, Yang Y, Ma W, et al. Ecosystem carbon stocks and their changes in china’s grasslands. Science China Life Sciences, 2010, 53: 757-765. https://doi.org/10.1007/s11427-010-4029-x

[11] Ni J. Carbon storage in grasslands of china. Journal of Arid Environments, 2002, 50: 205-218. https://doi.org/10.1006/jare.2001.0902

[12] Ni J. Carbon storage in terrestrial ecosystems of china: Estimates at different spatial resolutions and their responses to climate change. Climatic Change, 2001, 49: 339-358. https://doi.org/10.1023/A:1010728609701

[13] Zhang L, Zhou G, Ji Y, et al. Spatiotemporal dynamic simulation of grassland carbon storage in china. Science China Earth Sciences, 2016, 59: 1946-1958. https://doi.org/10.1007/s11430-015-5599-4

[14] Ma A, He N, Yu G, et al. Carbon storage in chinese grassland ecosystems: Influence of different integrative methods. Scientific Reports, 2016, 6: 21378. https://doi.org/10.1038/srep21378

[15] Xiao D, Deng L, Kim DG, et al. Carbon budgets of wetland ecosystems in china. Global Change Biology, 2019, 25: 2061-2076. https://doi.org/10.1111/gcb.14621

[16] Zheng Y, Niu Z, Gong P, et al. Preliminary estimation of the organic carbon pool in china’s wetlands. Chinese Science Bulletin, 2013, 58: 662-670. https://doi.org/10.1007/s11434-012-5529-9

[17] Guo M, Yang L, Zhang L, et al. Hydrology, vegetation, and soil properties as key drivers of soil organic carbon in coastal wetlands: A high-resolution study. Environmental Science and Ecotechnology, 2025, 23: 100482. https://doi.org/10.1016/j.ese.2024.100482

[18] Fang J, Liu G, Xu S. Soil carbon pool in china and its global significance. Journal of Environmental Sciences, 1996, 8: 249-254.
[19] Peng C, Apps MJ. Contribution of china to the global carbon cycle since the last glacial maximum: Reconstruction from palaeovegetation maps and an empirical biosphere model. Tellus B, 1997, 49: 393-408. https://doi.org/10.3402/tellusb.v49i4.15977
[20] Ji J, Huang M, Li K. Prediction of carbon exchanges between china terrestrial ecosystem and atmosphere in 21st century. Science in China Series D: Earth Sciences, 2008, 51: 885-898. https://doi.org/10.1007/s11430-008-0039-y
[21] Shao Q. Ecological conservation and restoration project in three-river source region boundary dataset [ds/ol]. National Ecosystem Science Data Center of China. 2022. https://doi.org/10.12199/nesdc.ecodb.aebknep.2022.003
[22] Shao Q. Natural forest protection project boundary dataset [ds/ol]. National Ecosystem Science Data Center of China. 2022. https://doi.org/10.12199/nesdc.ecodb.aebknep.2022.004
[23] Shao Q. Grain for green program boundary dataset [ds/ol]. National Ecosystem Science Data Center of China. 2022. https://doi.org/10.12199/nesdc.ecodb.aebknep.2022.005
[24] Shao Q. Returning grazing land to grassland project boundary dataset [ds/ol]. National Ecosystem Science Data Center of China. 2022. https://doi.org/10.12199/nesdc.ecodb.aebknep.2022.006
[25] Shao Q. Southwest china karst rocky desertification control project boundary dataset [ds/ol]. National Ecosystem Science Data Center of China. 2022. https://doi.org/10.12199/nesdc.ecodb.aebknep.2022.007
[26] Shao Q. Pearl river basin shelterbelt system construction project boundary dataset [ds/ol]. National Ecosystem Science Data Center of China. 2022. https://doi.org/10.12199/nesdc.ecodb.aebknep.2022.009
[27] Shao Q. Yangtze river basin shelterbelt system construction project boundary dataset [ds/ol]. National Ecosystem Science Data Center of China. 2022. https://doi.org/10.12199/nesdc.ecodb.aebknep.2022.008
